# A Feedback Quenched Oscillator Produces Turing Patterning with One Diffuser

**DOI:** 10.1371/journal.pcbi.1002331

**Published:** 2012-01-26

**Authors:** Justin Hsia, William J. Holtz, Daniel C. Huang, Murat Arcak, Michel M. Maharbiz

**Affiliations:** Department of Electrical Engineering and Computer Sciences, University of California, Berkeley, California, United States of America; University of Notre Dame, United States of America

## Abstract

Efforts to engineer synthetic gene networks that spontaneously produce patterning in multicellular ensembles have focused on Turing's original model and the “activator-inhibitor” models of Meinhardt and Gierer. Systems based on this model are notoriously difficult to engineer. We present the first demonstration that Turing pattern formation can arise in a new family of oscillator-driven gene network topologies, specifically when a second feedback loop is introduced which quenches oscillations and incorporates a diffusible molecule. We provide an analysis of the system that predicts the range of kinetic parameters over which patterning should emerge and demonstrate the system's viability using stochastic simulations of a field of cells using realistic parameters. The primary goal of this paper is to provide a circuit architecture which can be implemented with relative ease by practitioners and which could serve as a model system for pattern generation in synthetic multicellular systems. Given the wide range of oscillatory circuits in natural systems, our system supports the tantalizing possibility that Turing pattern formation in natural multicellular systems can arise from oscillator-driven mechanisms.

## Author Summary

The production of patterns in gene expression in an ensemble of cells is a phenomenon central to the development of multi-cellular organisms. Here we provide an exciting new result regarding diffusion-driven instability, a mechanism for spontaneous pattern formation originally proposed by Alan Turing. Efforts along this front have focused almost exclusively on Turing's original model and the “activator-inhibitor” models of Meinhardt and Gierer, but have yet to yield an experimental demonstration of a robust, tunable system that can break symmetry and spontaneously generate gene expression patterns. In this paper we propose a new family of oscillator-driven gene network topologies capable of Turing pattern formation. We believe this would be of significant impact to both emerging efforts at engineering multicellularity in the synthetic biology community as well as new guidance for those groups looking for similar phenomena in natural systems. Given the wide range of oscillatory circuits in natural systems, our system supports the tantalizing possibility that Turing pattern formation in natural multicellular systems can arise from oscillator-driven mechanisms. We provide an analysis of the system that predicts the range of parameters over which patterning should emerge and demonstrate the system's viability using stochastic simulations of a field of cells using realistic parameters.

## Introduction

Genetic networks which enable communication and coordination of behavior among cells in an ensemble have held the attention of developmental biologists and theoreticians [1]–[6] for over half a century. In particular, a vast body of literature – both theoretical [6], [7] and experimental [1]–[4] – exists which focuses on the production of patterns in gene expression, a phenomenon central to the development of multicellular organisms. A particularly well-studied mechanism for pattern formation is diffusion-driven instability, originally proposed by Turing [8], where a homogeneous steady state is destabilized in the presence of diffusion.

Recently, attempts have been made to build synthetic gene networks which generate spatio-temporal patterns in gene expression mediated by diffusible signals [9]–[13]. To obtain pattern generation, these efforts have relied either on the external spatio-temporal manipulation of the cell's chemical environment [9], [10], [13] or the precise positioning of cells containing different gene networks which secrete or respond to diffusible signals [11], [12]. To date, there have been no experimental demonstrations of a robust, tunable system which can break symmetry and spontaneously generate predictable gene expression patterns (spatio-temporal inhomogeneities) as in the Turing mechanism. What is specifically lacking in the community is an experimentally tractable model system for studying spontaneous pattern formation. Such a system would catalyze the engineering of complex cellular ensembles, ranging from engineered microbial communities [11], [13] to auto-differentiating multicellular systems.

**Figure 1:**
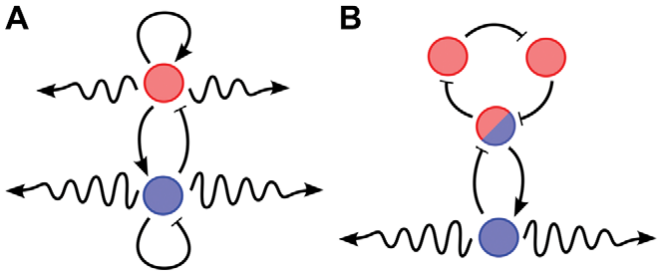
Comparison of architectures for Turing patterning. (A) The canonical “activator-inhibitor” system with activator in pink and inhibitor in blue. (B) The alternative “quenched oscillator” system. The *quenching loop* (in blue) with the diffusible molecule stabilizes the unstable *oscillator loop* (in pink). Diffusion then weakens the quenching loop's influence on the oscillator loop for spatial modes with high wave numbers and allows for the emergence of spatio-temporal oscillations.

In the synthetic biology community, efforts to achieve spontaneous generation of spatial patterns in gene expression have been centered around networks similar to the one originally proposed by Turing [8], and expanded into *activator-inhibitor* theory by Meinhardt and Gierer [5], [6], [14], [15]: two diffusible species interact with each other via chemical reactions that produce positive and negative interactions as in Figure 1A. For an appropriate range of kinetic parameters and diffusion constants, these topologies produce spatial or spatio-temporal patterns spontaneously from a homogeneous initial condition perturbed by small variations in concentration due to stochastic effects. However, this type of architecture has proven very difficult to implement using genetic networks because: (a) Turing instability requires that the steady state occur in the linear regime of the activator-inhibitor interactions away from saturation, and severely restricts the parameter range to meet the instability criteria; (b) when using systems with two diffusible components, either the diffusion constants [8] or the uptake rates [16] must be sufficiently different to allow unstable spatial modes, and significant differences are difficult to engineer; (c) the addition of intermediate protein steps to two-molecule activator-inhibitor models further restricts the parameter set for patterning; and (d) stochasticity plays a significant role in the behavior of these systems, but most analyses rely on continuum partial differential equation (PDE) models, making it difficult to reconcile theoretical predictions with observed experimental results.

Although the activator-inhibitor model is the canonical example of a system demonstrating Turing instability, many other possible network structures exist. Indeed, the essential structural requirement for the emergence of the Turing phenomenon is that the network contain an unstable subsystem, which is stabilized by a feedback loop. The diffusion of molecules participating in this feedback loop then unleashes the inherent instability and allows growth of spatial modes. In the activator-inhibitor network in Figure 1A, the activator plays the role of the unstable subsystem and the inhibitor provides the stabilizing feedback. Although it is well known that the Turing mechanism is not restricted to the activator-inhibitor network (see, e.g., [17] for Turing instability conditions for general reaction-diffusion models), to the best of our knowledge, no other biologically plausible network has been proposed. Systems that contain more than two species have been studied, but their reactions conform to the essential structure of the activator-inhibitor paradigm [6].

This paper breaks away from the activator-inhibitor model and proposes a new network which we call a “quenched oscillator” system. This system uses one diffusible component and an oscillator circuit serving as the unstable subsystem that is quenched by a second feedback loop, as depicted in Figure 1B. To our knowledge, this is the first demonstration that oscillator-driven gene networks can exhibit Turing instability and spatial patterning of gene expression across fields of cells. Moreover, the network can be implemented with a variety of published oscillator circuits [18]–[20] using known genes and promoters.

It is important to stress that the mechanism pursued here – Turing instability – is fundamentally different from the traveling wave trains and spiral waves in diffusively coupled oscillators [7], [21]. The proposed architecture bears resemblance to the diffusively coupled repressilator model in [22], where a second loop is integrated with the repressilator to incorporate a diffusible molecule, and the diffusively coupled oscillator model in [23]. However, in both of these systems, the oscillator is not quenched, but is simply allowed to communicate between cells to ensure synchronization, which is contrary to the pattern formation task studied here. Although we employ an oscillator as a subsystem, the full system in isolation is not an oscillator, instead exhibiting a stable steady state as in the Turing mechanism, and is fundamentally different from out-of-phase oscillator systems.

The patterns presented in this paper are oscillatory in both time and space (see Text S2). While they still fall under the category of diffusion-driven instability as proposed by Turing, some researchers associate the term “Turing patterning” with stationary spatial non-uniformities [24]. However, in the remainder of this paper, the use of the term “Turing pattern” incorporates oscillatory Turing patterns.

**Figure 2:**
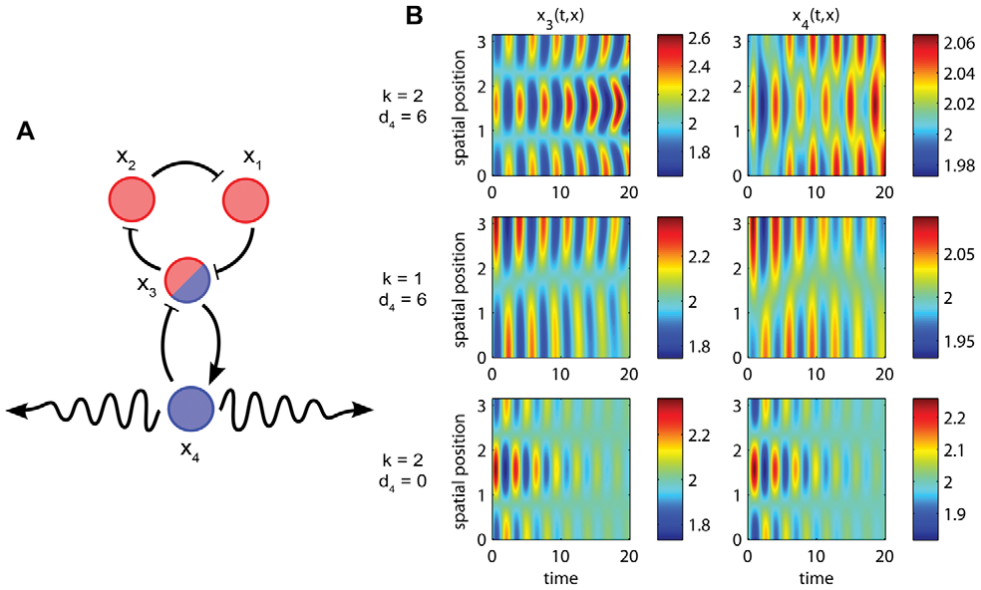
PDE simulation results for the toy model. The system (1) run with parameters 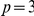, 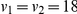, 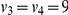, 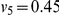, and 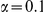. Position, time, and concentrations are scaled to be dimensionless. See Figures S1, S2 for full simulation results. (A) Toy model network with labeled species. Coloring scheme for loops follows that of Figure 1B. (B) With 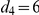, Turing instability conditions are met for a slight perturbation of the homogeneous initial condition with the second wave (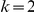), and growth of the inhomogeneity follows (top row). Solution 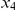 looks qualitatively different than 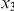 and the other species due to the “bleeding” effect of diffusion. With 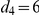, Turing instability conditions are not met for 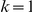 and the initial inhomogeneity decays slowly in time (middle row). With 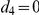, the initial inhomogeneity 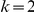 decays in time (bottom row).

Systems which produce oscillating patterns have previously been reported [22], [23], [25], [26], but there are fundamental differences between these systems and our own. In Turing's diffusion-driven instability, the biological system exists in a population of homogeneous cells. In isolation, each individual system is stable and reaches a steady state over time. In the presence of diffusion, the steady state is destabilized and spatial inhomogeneities arise. The systems presented in [22], [23], and Figure 2C of [25] are not stable individually, so the patterns they produce do not fall under the general category of “Turing patterns.” The systems presented in [26] and Figure 2A and 2B of [25] are stable, but do not contain an oscillatory subsystem. Our system utilizes an oscillator subsystem, which does not constitute positive feedback; this is a crucial point that separates it from previous systems.

In addition to the new architecture presented in this paper, we believe the methodology used to find and tune new pattern-generating systems may prove of significant value to practitioners. We recognize that Turing patterning is just one possible method to achieve the generation of gene patterning across a population of cells, but it is a phenomenon that is well-characterized mathematically, allowing us to develop the combined PDE/stochastic simulation approach presented here. Patterning has proven difficult to produce experimentally, so these analysis tools should aid in the search for more reliable experimental systems.

Below, we first provide an analysis of the system which predicts the range of kinetic parameters over which patterning should emerge. We show the architecture produces patterning for parameters within the range of values present in the literature for our molecules. Using both continuous, deterministic simulations (henceforth called “PDE simulations”) and discrete, stochastic simulations (henceforth called “stochastic simulations”) of fields of cells, we demonstrate how stochastic molecular interactions affect pattern formation in the limit of very low concentrations of molecular species per cell. The primary goal of this paper is to provide a circuit architecture which can be implemented with relative ease by practitioners and which provides an alternative implementation strategy for reaction-diffusion pattern generation in synthetic multicellular systems. Lastly, given the wide range of oscillatory circuits in natural systems, our system supports the tantalizing possibility that Turing-like pattern formation in natural multicellular systems can arise from oscillator-driven mechanisms.

## Results

### The quenched oscillator system permits Turing phenomena

The first feedback loop in our design is an oscillator. The second feedback loop is designed to quench these oscillations, meaning that, in the presence of the second loop, the first loop ceases to oscillate and the full system instead approaches a steady-state solution. If the oscillator design is based on a phase lag mechanism as in Figure 1B, then it is essential that the second loop with the diffusible molecule (in blue) have smaller phase lag than the first loop (in pink), so that it is stable by itself and that it stabilizes the oscillator when interconnected. Smaller phase lag can be achieved with fewer reaction steps or with faster degradation rates in the second loop.

As an illustrative example, consider the following “toy” model, possessing both an oscillator loop 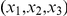 and a quenching loop 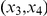:
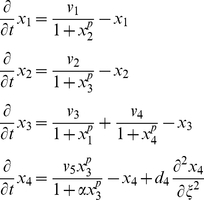
(1)where the concentrations 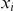, 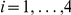, and all other variables and parameters are non-dimensional. In particular, the time variable 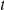 is scaled to bring the degradation constants (assumed to be identical for each species for simplicity) to one, and the one-dimensional length variable 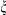 is scaled so that the spatial domain is 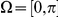. We assume only the fourth species is diffusible (represented with wavy arrows in Figure 1B) with diffusion coefficient 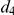 and is subject to zero-flux boundary conditions, meaning there is no diffusion at the ends of the line of cells at 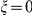 and 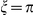.

For analysis, we take the linearized form of the reaction-diffusion system above:
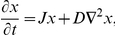
where 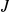 is the Jacobian matrix of the vector field of reaction rates evaluated at the steady state of the reaction system, 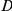 is the diagonal matrix of diffusion coefficients, and 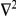 is the vector Laplacian. For our toy model, the Jacobian matrix about the steady state 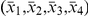 is:
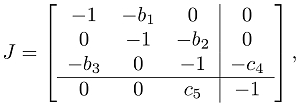
(2) where:
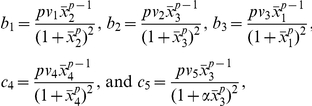
and 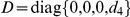. The dynamical behavior of this reaction-diffusion system is determined from the matrices 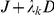, where 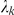 are the eigenvalues of the Laplacian operator 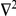 on the given spatial domain, and the subscripts 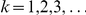 denote the wave numbers. On our one-dimensional domain 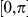, 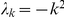 and the eigenfunctions are the cosine waves 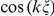 [27]. If the matrix 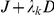 is stable (that is, if all of its eigenvalues have negative real parts), then the corresponding spatial wave decays to zero asymptotically in time. If 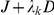 is unstable (at least one of its eigenvalues has positive real part), then the corresponding spatial wave grows.

Let 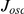 be the upper-left 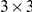 submatrix of 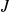, corresponding to the oscillator loop. For diffusion-driven instability to arise in this network, the following three conditions must be met:

**Condition 1.** The oscillator loop by itself would produce oscillations (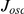 is unstable).

For the oscillator subsystem to be unstable, we need:
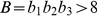
(3)so that the characteristic polynomial of 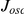, given by 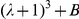, has a pair of complex conjugate roots with positive real part.

**Condition 2.** The quenching loop ceases oscillations in the full system (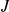 is stable).

For stability of the full reaction network, we need:
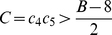
(4)so that 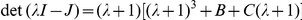 has all roots with negative real parts.

**Condition 3.** Diffusion will weaken the quenching loop's influence on the oscillator loop for high wave numbers, allowing spatio-temporal oscillations to emerge (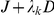 is unstable for some 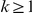).

For diffusion-driven instability of the 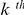 spatial mode 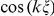, the polynomial:
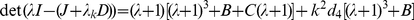
(5)must have at least one eigenvalue with positive real part. Indeed, when the product 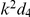 is sufficiently large, three roots of (5) approach those of 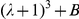, which contain roots with positive real part due to (3). This means that the inhomogeneous modes 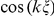 grow over time if 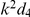 exceeds the threshold for instability of the polynomial (5), which we will call 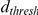. This implies that, for diffusion-driven patterning, we need a large diffusion coefficient or a large wave number. More generally for 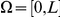, we need 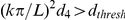, meaning patterning can also be achieved for a small spatial domain. See Text S1 for details.

The parameters 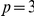, 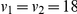, 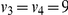, 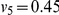, and 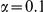 in the system (1) satisfy conditions (3) and (4) with 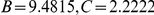. The polynomial (5) becomes unstable when 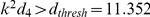. PDE Simulations with 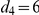 indeed exhibit growth of the spatial inhomogeneity when the steady state is perturbed by adding the second wave (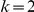) with amplitude steady state 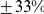 peak-to-peak to 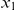 (Figure 2B, top). The PDE system does not include noise, so a perturbation must manually be added to the system for cells to leave the steady state. This Turing behavior is contrasted to the decay of the initial inhomogeneity for wave numbers below the instability threshold (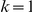, 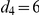 in Figure 2B, middle) and in the absence of diffusion (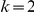, 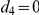 in Figure 2B, bottom).

### A quenched oscillator system can be designed using existing oscillators

**Figure 3:**
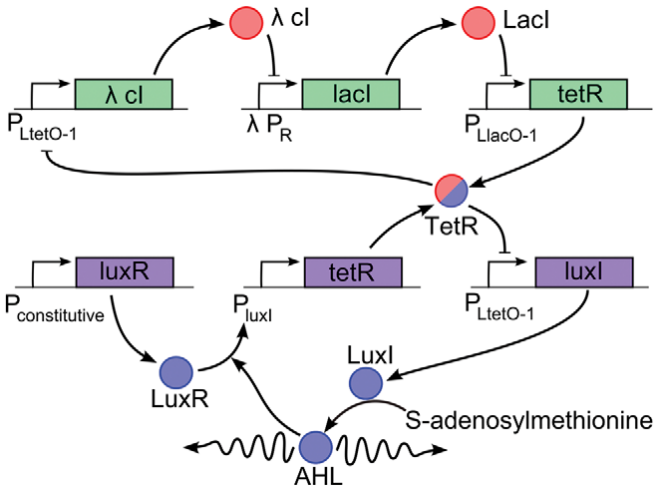
A possible synthetic implementation of the network in **Figure 1B**. Two feedback loops are interconnected by shared production and sensing of the transcriptional repressor *tetR*. The oscillator loop is represented by the green genes and pink molecules and the quenching loop is represented by the purple genes and blue molecules. The second loop contains the membrane-diffusible signaling molecule acyle-homoserine lactone (AHL).

We now propose a novel network that can be synthesized from existing components. Consider the system of two interconnected loops shown in Figure 3. The first (top) loop is the repressilator [18], which is a ring oscillator, comprised of three pairs of transcriptional repressors (TetR, 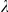 cI, LacI) and promoters 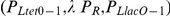, which match up with the three-component oscillator of the toy model (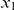-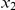-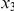). The second (bottom) feedback loop consists of *V. fischeri* quorum sensing genes *luxI* and *luxR*. The *luxI* gene is regulated by the 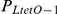 promoter, and is transcribed in the absence of TetR. LuxI is the synthetase that catalyzes the formation of the membrane-diffusible signaling molecule acyl-homoserine lactone (AHL). AHL binds to the constitutively produced protein, LuxR. The LuxR-AHL complex forms a homodimer that binds to the 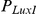 promoter and activates transcription. TetR production closes the second loop by repressing the second 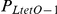 promoter. This quenching loop is much longer than that of the toy model (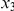-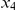), but still contains a single diffusible molecule, AHL, and we ensure that it has smaller phase delay than the oscillator loop by using faster degradation rates. Even though the bottom loop has a single inhibitory interaction, this loop does not oscillate because the phase delay is small. The two loops interact through TetR and the first loop ceases to oscillate in the presence of the second loop.

### Constraints on kinetic and diffusive parameters can be obtained from a PDE model

We represent the dynamics of the network in Figure 3 with the following set of partial differential equations:
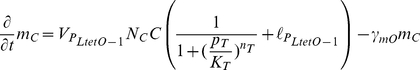
(6)

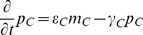
(7)

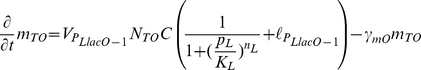
(8)

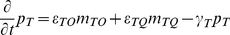
(9)

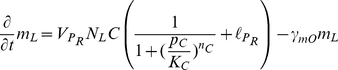
(10)

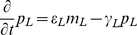
(11)

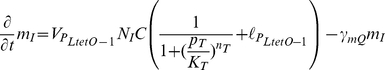
(12)

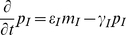
(13)

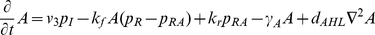
(14)

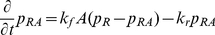
(15)

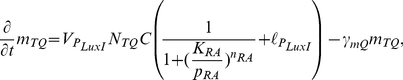
(16)where 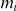 are mRNA concentrations, 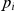 are protein concentrations, 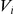 are velocity constants, 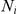 are copy numbers, 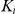 are dissociation constants, 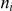 are Hill coefficients, 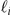 are leakage rates normalized to 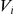, 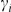 are degradation rates, and 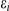 are protein translational rates. The parameters are subscripted according to their corresponding species (*C* = [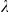 *cI*], *T* = [*tetR*], *L* = [*lacI*], *I* = [*luxI*], *A* = [*AHL*], *R* = [*luxR*], *RA* = [*luxR-AHL* complex]) except for velocity and leakage constants, which are subscripted by promoter, and copy numbers, which are subscripted by the gene being transcribed. The concentration of the mRNA for *tetR* is split into those produced by the oscillator loop (*O*) and the quenching loop (*Q*). The variable 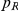 is the total amount of LuxR protein in the system, which is assumed constant, thus the amount of free LuxR is represented by 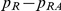. The parameter *C* is the concentration level generated by a single molecule in an *E. coli* cell and 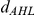 is the diffusion coefficient of *AHL*. We take 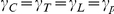. The system is subject to zero-flux boundary conditions on the one-dimensional spatial domain 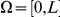.

Following the same procedure as outlined for the toy model, we solve for constraints on kinetic and diffusive parameters by deriving expressions used to satisfy the three conditions for Turing instability (see Text S1). These expressions are complex combinations of the many parameters in our system, but still reveal ways of manipulating parameter choices to meet the Turing instability conditions. In this analysis, the protein translation rates were taken to be the most readily tunable, which made the big challenge finding protein translation rates for this system to meet the Turing conditions for patterning.

### Experimentally reasonable parameter sets produce spatio-temporal patterning

**Figure 4:**
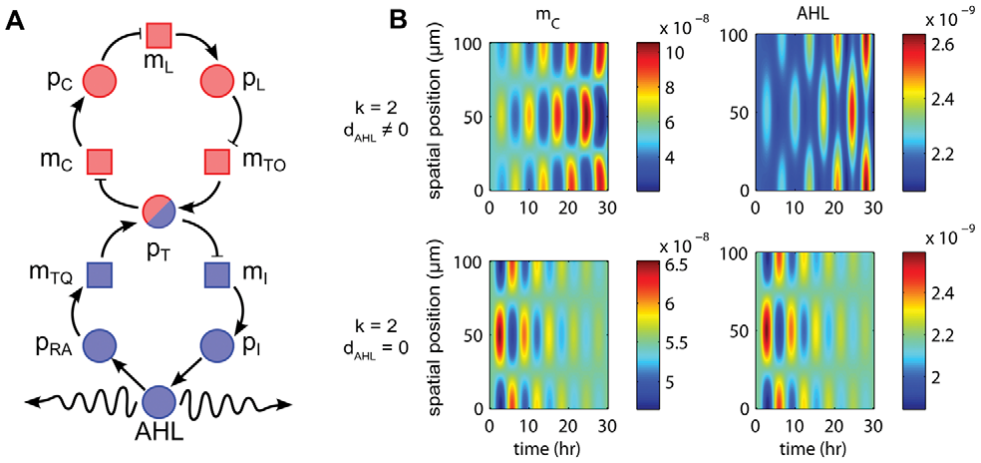
Representative sample of PDE simulation results using Parameter Set 1. Concentrations (colorbar) given in 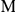. The behavior of 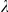 cI mRNA (left) is qualitatively similar to that of 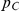, 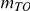, 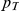, 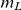, 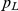, 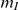, and 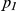 and the behavior of AHL (right) is qualitatively similar to that of 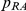 and 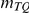. See Figures S3, S4, S5 for full simulation results. (A) Quenched oscillator network with labeled species. Here squares represent mRNA and circles represent proteins and other molecules. Color scheme for loops follows that of Figure 1B. (B) With 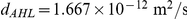, the imprinted wave 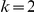 grows (top row). With 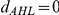, the imprinted wave decays (bottom row).

To show the viability of this system for experimental implementation, we modeled the system behavior using parameter values from the literature that fit the constraints found in the analysis (“Value for PDE Simulation (Parameter Set 1)” column of Table S1). Expected steady-state values and instability measurements can be found in Tables S2 and S3. We ran PDE simulations in MATLAB with and without AHL diffusion using an initial perturbation in 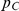 of amplitude steady state 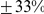 peak-to-peak and wavelength 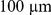, which was predicted to be unstable (Figure 4). The imprinted wave grows with diffusion and it decays without diffusion, exhibiting similar behavior to that of the toy model (Figure 2).

**Figure 5:**
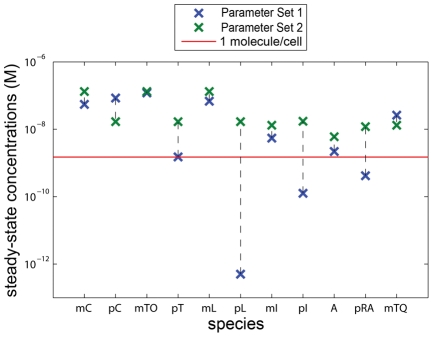
Comparison of steady-state concentrations for Parameter Sets 1 and 2. A number of steady-state concentrations for Parameter Set 1 lie near or below the threshold of 1 molecule/*E. coli* cell (red line). Parameter Set 2 has been chosen such that all steady-state concentrations lie above this threshold.

While the simulation results produce spatio-temporal patterning as desired, the expected experimental behavior will be impacted by stochastic properties that stem from concentrations in our system approaching a few molecules per cell. Taking the concentration of a single molecule in an *E. coli* cell to be 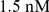 [28], a number of steady-state values fall near or below this threshold (Figure 5), particularly 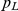 and 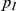. This implies that: a) stochastic simulations are necessary for examining experimental plausibility, and b) Parameter Set 1 would need to be modified to produce pattern due to the behavior of certain species in our system being dominated by noise. In this limit, stochastic models better capture the behavior of *in-vivo* systems because of their inability to respond to aphysical concentration changes of less than one molecule per cell.

**Figure 6:**
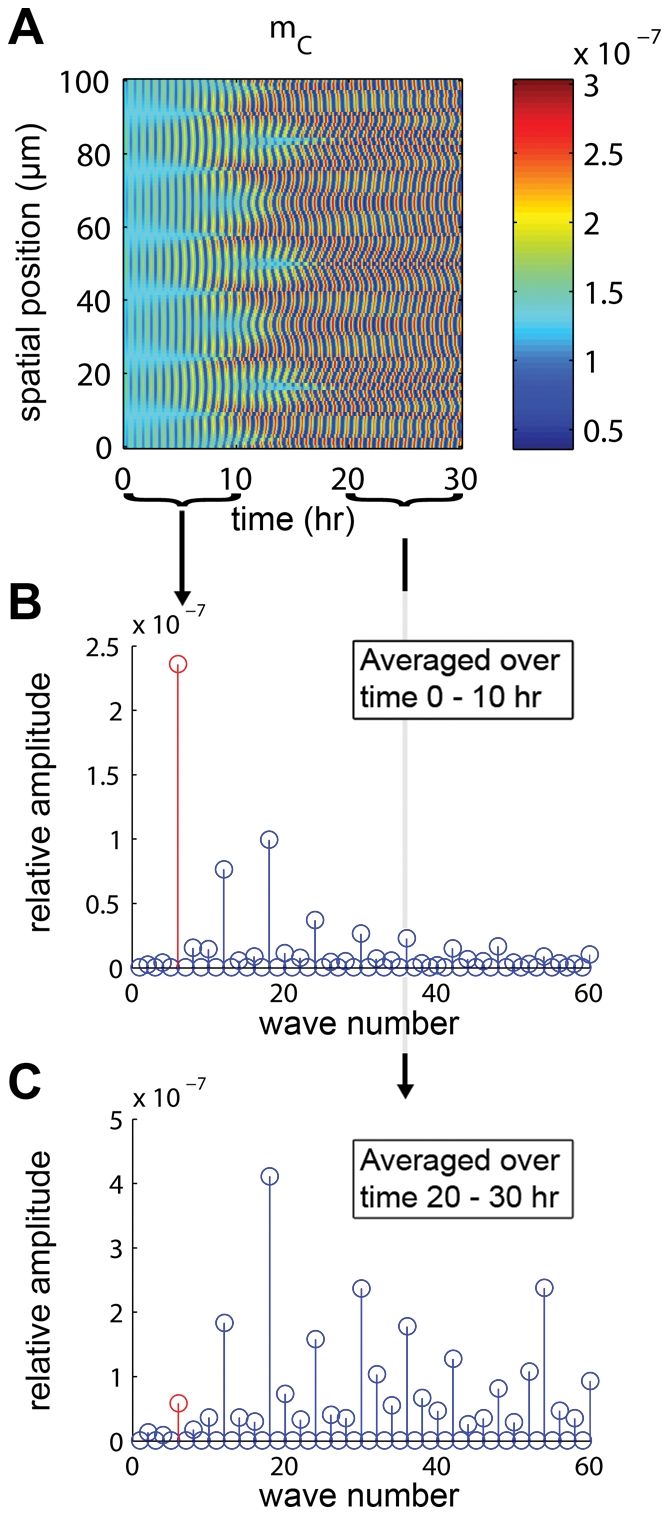
Results and analysis of PDE simulation for Parameter Set 2. (A) PDE simulation results for 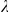 cI mRNA using Parameter Set 2 in Table S1 to draw comparisons between parameter sets (Figure 4B). Concentrations (colorbar) given in 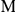. The imprinted wave 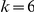 was chosen because it falls above the minimum unstable wave number of 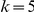 for this parameter set (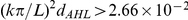). Parameter Set 2 oscillates much faster than Parameter Set 1, and because of this we observe more interesting behavior within the 30-hr simulation window once the oscillations reach their maximum amplitude. In particular, the imprint initially grows, but then the energy moves into higher harmonics as time goes on. See Figure S6 for full simulation results. (B) Discrete cosine transform (DCT) of 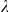 cI mRNA over the window of 0–10 hr. The imprinted wave (shown in red) dominates and grows. (C) Over the window 20–30 hr, higher harmonics have begun to dominate.

Given complete freedom in choosing parameter values, our analysis would allow us to methodically identify regions in the parameter space that should produce patterning. It is encouraging that even when restricting ourselves to literature values for all of the parameters, we were able to demonstrate spatio-temporal patterning in PDE simulation. Here we show that with other parameter values that are still biologically realistic (“Value for Stochastic Simulation (Parameter Set 2)” column of Table S1), we can improve the system performance to also produce patterning in a discrete, stochastic environment. All of these values are physically possible based on information in the literature (see references in Table S1). To accommodate the change in the ratio 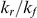, LuxI has been replaced in our system with the AHL synthetase RhiI from *P. aeruginosa*. A more thorough explanation of the origin of these two parameter sets can be found in Text S3 and Text S5. As more biological parts are characterized or created, parts are likely to be found that match our chosen parameter values. The steady-state concentrations for Parameter Set 2 can also be seen in Figure 5 and do not fall below 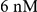 (4 molecules/cell). We also verified the desired system behavior of this parameter set in PDE simulation (Figure 6). This new parameter set results in growth of additional wave numbers other than the imprinted one, highlighting the nonlinear nature of our system. These effects arise when oscillations start to reach near-maximal amplitudes and would likely be seen for Parameter Set 1 if the simulations were run for a much longer time.

### Stochastic simulations confirm the emergence of patterning

We developed a set of reactions for stochastic simulation that, using the law of mass action and the quasi-steady-state approximation, would exactly match our set of PDEs. The full set of reactions used in our stochastic simulations can be found in Text S4.

**Figure 7:**
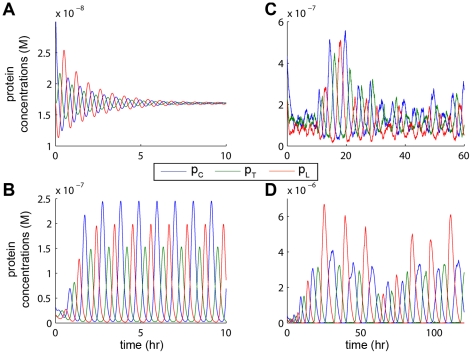
Comparison of PDE and stochastic simulations in a single cell using Parameter Set 2. Stochastic simulations exhibit more irregular and slower oscillations, due to stochasticity and our modeling assumption that the dimerization and binding reactions are at equilibrium in the PDE model, but the species follow the same phase relationships ( spike, 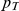 spike, then 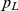 spike). See Figures S7, S8, S9, S10 for full simulation results. (A) PDE simulation with no diffusion is stable with asymptotically decaying oscillations. (B) PDE simulation with AHL diffusing away quickly reaches an extremely regular limit cycle. (C) Stochastic simulation with no diffusion exhibits small amplitude oscillations. Occasionally a “firing event” will occur, as seen around hour 10, where the system will grow briefly before settling again. (D) Stochastic simulation with AHL diffusing away shows oscillations with irregular amplitudes, but much larger than those of the non-diffusing case. In both the PDE and stochastic simulations, the period of oscillations is longer with diffusion than without.

To compare the behavior of PDE and stochastic simulations, we first ran single cell simulations to verify that the general expected behavior was maintained. While not indicative of the system's ability to generate pattern, these simulations allow us to draw comparisons between our PDE and stochastic models. To observe both an oscillating cell and a quenched cell, we used a single cell in the center of a long, empty volume. Without AHL diffusion, the cell remains isolated and we expect oscillations to decay to the steady state. With diffusion, AHL diffuses into the empty volume and weakens the quenching loop, meaning oscillations are expected to grow. Both PDE and stochastic simulations confirmed these expectations (Figure 7). The simulations exhibited similar behavior but oscillations in the stochastic environment are slower and more irregular, due to stochasticity and our modeling assumption that the dimerization and binding reactions are at equilibrium in the PDE model. Oscillations in the stochastic simulations are significantly slower – about 5 times slower in the decaying case, and 10 times slower in the growing case – which lead us to choose faster degradation rates for Parameter Set 2. In a cell without diffusion, stochasticity keeps the system oscillating at a small amplitude with occasional “firing events,” where a few cycles of increased oscillation amplitude occur before the system settles again. Both PDE and stochastic simulations exhibit the same phase relationship between the proteins in the oscillator loop and a slower period of oscillation when growing as opposed to decaying (Figure 7 and S7, S8, S9, S10).

As expected, stochastic simulations with Parameter Set 1 in a line of cells were unable to produce patterning due to the low steady-state concentration values (results not shown), but did yield some insights. In particular, any initial imprint we imposed would very rapidly (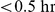) decay into noise, likely due to low copy numbers. With only four or five promoter binding sites per cell and the fact that almost all of them are bound in steady state, a large change in a single species of the system is unlikely to be able to propagate quickly enough throughout the system due to the bottlenecks at the promoter binding sites. Thus we avoided imprinting and used the ability of the stochasticity in our system to naturally excite high wave numbers.

**Figure 8:**
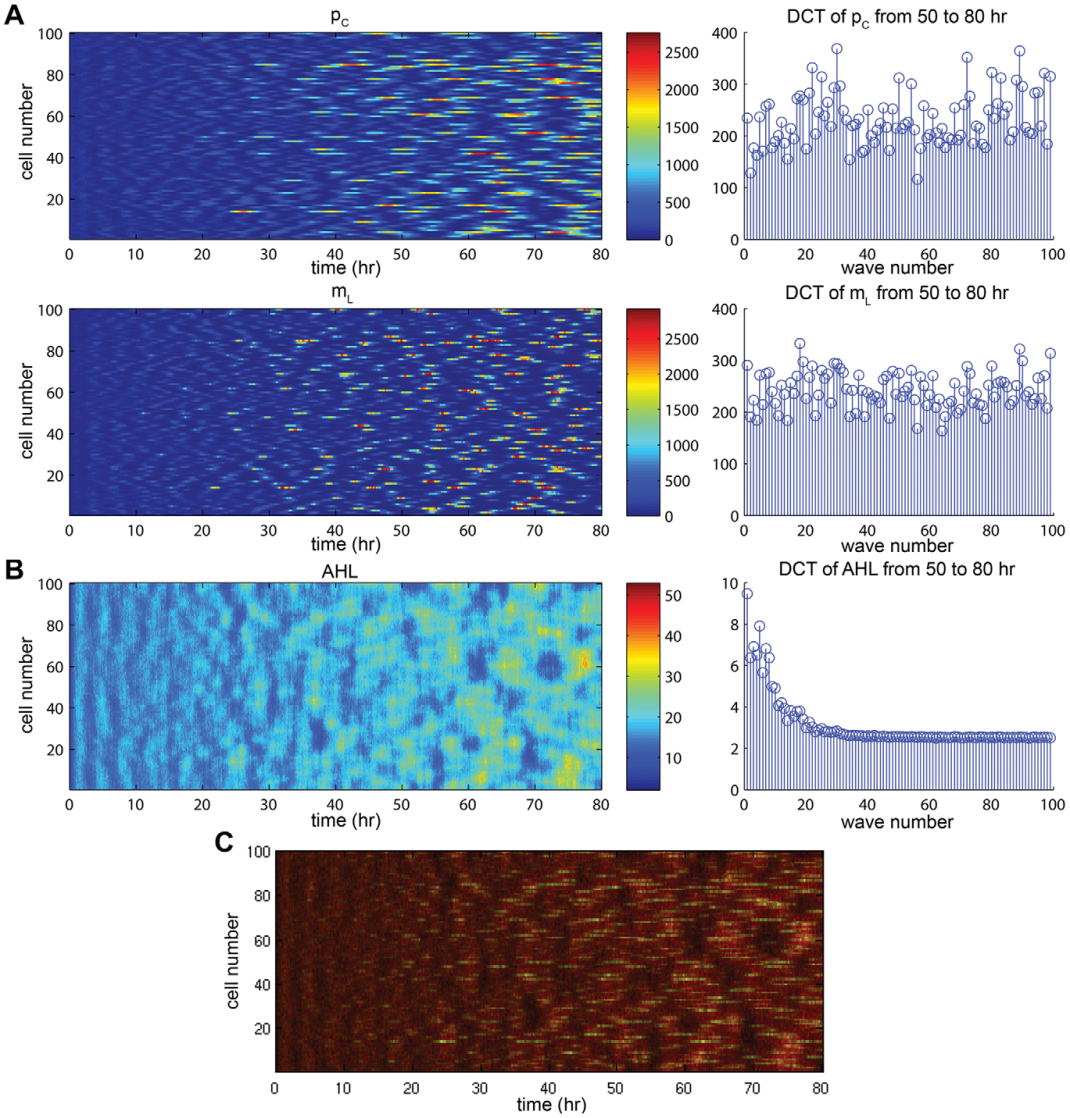
Representative sample of stochastic simulation results for a line of cells with homogeneous initial condition. Included are color plots (left) and DCTs averaged over hours 50 to 80 (right). The stochasticity causes oscillations to arise naturally and can be seen as early as hour 20. See Figures S11, S12 for full simulation results. (A) Results for 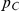 (top) and 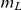 (bottom) are indicative of the behavior for mRNA and proteins for 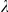 cI, TetR, LacI, and LuxI. While the DCT plots vary from species to species, certain wave numbers are found to be more pronounced across all species, particularly 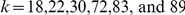. (B) Results for AHL produce similar behavior in all downstream species in the quenching loop. Both the color plot and DCT are markedly different due to the effects of diffusion, which causes a “spreading” of the rapid peaks seen in mRNA and protein color plots and acts like a low-pass filter in the frequency domain. (C) Overlay plot of AHL and 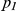 demonstrating the correspondence between the peaks in the species as well as the effect of diffusion. AHL was monochromed in red and 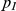 in green, leading to the appearance of yellow in areas of large overlap.

Indeed, stochastic simulations with Parameter Set 2 in a line of cells exhibit growing oscillations and eventually produce spatio-temporal patterning (Figure 8). Large amplitude oscillations emerge around 20 hours and an obvious pattern emerges as time goes on. Visually, patterning is most evident in AHL due to the effects of diffusion. Without diffusion, no spatial patterns emerge with single cell oscillations occurring randomly (results not shown).

To quantify the patterns produced by our system, we use the *discrete cosine transform* (DCT) to check the relative presence of the different emerging wave numbers. All wave numbers higher than a threshold (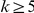 for Parameter Set 2) should grow in the presence of noise according to our analysis, but a number of factors, including stochasticity and the discrete nature of only having 100 cells in our simulations, prevent them from growing uniformly. The exact wave numbers vary from simulation to simulation, but the averaged DCT over time frames late in simulations (beyond the “start-up” phase) always shows a number of spikes that are prominent across most species in the system (see Figures S11, S12). The exceptions to this are AHL and subsequent species in the quenching loop, where diffusion acts as a low-pass filter and attenuates high wave numbers. This filtering effect is what accounts for the visual “bleeding” effect of diffusion.

## Discussion

### Parameter space and chosen parameter sets

The process of producing a set of parameters which produce pattern in the stochastic regime provided several insights which can inform implementation decisions as new promoters, proteins, and parameter manipulation techniques become available. These findings may also be of use when searching for putative natural systems which exhibit this behavior.

Two of the most restrictive parameters that we had to change significantly from our initial solution set were promoter leakage rates and dissociation constants. High amounts of leakage makes it simultaneously more difficult to make the oscillator subsystem unstable and more difficult for the quenching loop to stabilize the overall system. The dissociation constants directly affected the steady-state concentrations of the protein species in our system; the system fails to produce patterning when these values are too small. These observations were made from studying the form of the expressions for 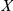 and 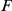 (see Text S1) and many other such observations and insights can be drawn from the analysis.

A few considerations only became relevant when performing stochastic simulations, the biggest of which was the bottleneck of promoter binding sites. In the PDE model, new mRNA would be produced at a rate that was a function of the amount of the appropriate activator or inhibitor in the system. By enumerating the number of promoter binding sites, we decrease the sensitivity of the system to very large concentrations of the activators and inhibitors and increase the importance of each binding and unbinding event. Analytically, we can maintain the same system behavior by holding the product 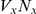 in each mRNA differential equation constant. Arbitrarily increasing the copy numbers this way has its own drawbacks. We assume the concentration of LuxR is constitutively produced and is constant. At our current value of 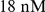 (12 molecules/cell), we can only bind at most six promoters with LuxR-AHL dimers, so having a large 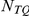 will not change the amount of 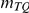 being produced, which deviates from what our PDE model predicts.

### Experimental plausibility

Assuming proper parameter values can be chosen for our system, our analysis generates a testable hypothesis for a possible experimental implementation. When setting up the experiment, the following additional concerns should be taken into account.

Beyond finding parameters that meet the Turing instability conditions, system speed is very important because it determines the visibility of changes in the system over the course of a normal experiment duration. System speed is most directly affected by the degradation rates of every species in the system. These change the period of oscillations as well as the growth and decay rates of wave modes. Very slow growth and decay would delay the emergence of visible patterns and make experimental debugging difficult because any activity would be hard to observe. Very long experiments are problematic in terms of collecting data and dealing with cell division and lifespan.

A reporter gene was unnecessary in simulation, but one would need to be used in experiments. As seen in Figure 8, there are two distinct types of qualitative behaviors: the proteins 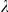 cI, LacI, TetR, and LuxI exhibit brief bursts localized to single cells while AHL and subsequent quenching loop species exhibit more spread out behavior due to diffusion. It is possible to attach a fluorescent protein to the appropriate loop to follow either type of behavior. While AHL may produce a more visually-pleasing patterning, the oscillator loop species undergo larger swings in number of molecules, which would be easier to discern in units of fluorescence.

### Implications of the novel architecture

The engineering of cooperative ensembles of cells, whether in the context of designer microbial communities or other synthetic multicellular systems will require tractable model systems which exhibit spontaneous symmetry breaking and pattern formation, both fundamental prerequisites for any kind of replicating or “programmed” heterogeneity of form or function. Attempts to produce spontaneous pattern formation using Turing's canonical system have proven difficult (see Introduction). This paper breaks away from the activator-inhibitor model and alleviates some of the difficulties encountered by using oscillating subsystems. To our knowledge, this is the first attempt of this kind and significant effort was devoted to providing researchers with an experimentally tractable road map towards implementation.

This work also implicitly suggests that natural systems may have arisen where oscillating subsystems, initially evolved for other purposes, provide the backbone not just for coordinated oscillation (as in the diffusively coupled systems demonstrated by others [7], [22], [23]) but for robust Turing-type pattern formation phenomena. It is not difficult to find examples in the recent literature of naturally-occurring coupled negative feedback oscillators, both in prokaryotes [29] and eukaryotes [30], [31]. A function as fundamental as cell cycle oscillation appears to be maintained in yeast and other eukaryotes by coupled oscillators (a negative feedback oscillator coupled to a relaxation oscillator) [31]. Going further, these motifs are also present in protein-protein systems [32]; while outside the scope of the present work, the general results presented (i.e. coupled multi-step negative feedback oscillators with one diffusible component can exhibit Turing instability) would likely apply to kinase loops [32]. Lastly, in our model the relative phase lag between the oscillator loop and the quenching loop affect both the emergence and wave numbers of pattern; these, in turn, depend on the relative number of “steps” around the loops. It is tempting to suggest that the alteration of the number of steps, or the total delay around the loop, could provide a mechanism by which adaptation and evolution could generate systems (and variants) capable of pattern formation.

## Materials and Methods

### PDE simulations

Continuous, deterministic models are useful because of the wide variety of analysis tools we can apply to them to generate predictions of system behavior and workable parameter spaces, which we cannot do for stochastic models. These models are accurate when the number of molecules for all species in the system are very large, but generally need to be supplemented with stochastic simulations for systems with small numbers of molecules. PDE simulations were run in MATLAB Version 7.10.0.499 (R2010a) with the function ode15s, which is a multi-step, variable order solver based on numerical differentiation formulas. For line of cell simulations, diffusion was handled using a finite difference approximation with 101 evenly-spaced grid points and zero-flux boundary conditions. For single cell simulations, the long empty volume was represented using a finite difference approximation with Dirichlet boundary conditions of zero AHL concentration.

### Stochastic simulations

Stochastic simulations of the network were performed using the Stochastic Simulator Compiler (SSC) v0.6 [33]. The output from SSC was reformatted with custom Perl scripts and then plotted in MATLAB. SSC handles concentrations in units of molecules, so all parameter values were scaled appropriately, but the output values were converted to units of molarity in the figures given in this paper for ease of comparison. Reported values for protein concentrations are the totals of all forms of the protein: monomer, dimer, and bound to promoter. We represented cells with cubes of edge length 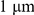. For single cell simulations, the cell was located at the center of a volume of 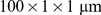. All multi-cell simulations consisted of a line containing 100 directly adjacent cells.

### Discrete cosine transforms

A discrete cosine transform (DCT) expresses a finite sequence of data as a sum of cosine functions of different frequencies [34]. The eigenfunctions of the Laplacian operator on a one-dimensional spatial domain with zero-flux boundary conditions are cosine functions [27], which are represented more accurately by the DCT than by the discrete Fourier transform, which is appropriate for periodic boundary conditions. The DCT is useful for our analysis because it allows us to examine the presence of certain spatial wave numbers in a line of cells simulation relative to the other wave numbers and how these relations change over time. Because the amplitudes of a DCT are changing in time and can be both positive and negative, we take the average of the absolute values of spatial DCTs over an interval of time. This was handled in MATLAB using the function dct. Because concentrations are non-negative, there is always a significant offset component 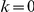, which we omit from our figures for better scaling of the remaining wave numbers.

## Supporting Information

Figure S1
**PDE simulation results for toy model in line of cells with diffusion.** Here 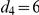 and 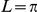. Position, time, and concentrations are scaled to be dimensionless. Perturbation in 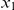 of amplitude steady state 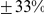 peak-to-peak. (A) When 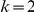 (wavelength 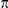), 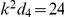 falls above the instability threshold of 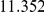 and the inhomogeneity grows. (B) When 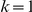 (wavelength 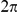), 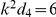 falls below the instability threshold of 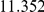 and the inhomogeneity decays.(TIFF)

Figure S2
**PDE simulation results for toy model in line of cells without diffusion.** Here 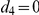, 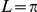, and 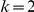 (wavelength 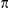). Position, time, and concentrations are scaled to be dimensionless. Perturbation in 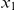 of amplitude steady state 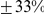 peak-to-peak. The cells don't communicate and each one is stable, so the inhomogeneity decays.(TIFF)

Figure S3
**PDE simulation results for Parameter Set 1 in line of cells with diffusion and unstable wavelength.** Here 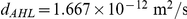, 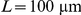, and 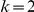 (wavelength 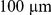). Concentrations (colorbar) given in 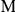. Perturbation in 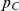 of amplitude steady state 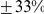 peak-to-peak. The inhomogeneity grows.(TIFF)

Figure S4
**PDE simulation results for Parameter Set 1 in line of cells with diffusion and stable wavelength.** Here 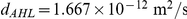, 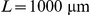, and 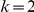 (wavelength 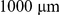). Concentrations (colorbar) given in 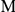. Perturbation in 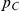 of amplitude steady state 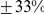 peak-to-peak. The inhomogeneity decays. To achieve a stable wavelength (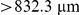), we had to increase the spatial domain.(TIFF)

Figure S5
**PDE simulation results for Parameter Set 1 in line of cells without diffusion.** Here 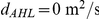, 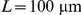, and 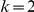 (wavelength 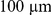). Concentrations (colorbar) given in 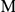. Perturbation in 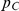 of amplitude steady state 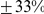 peak-to-peak. The inhomogeneity decays.(TIFF)

Figure S6
**PDE simulation results for Parameter Set 2 in line of cells with diffusion.** Here 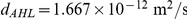, 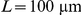, and 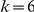 (wavelength 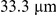). Concentrations (colorbar) given in 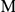. Perturbation in 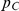 of amplitude steady state 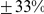 peak-to-peak. The inhomogeneity grows.(TIFF)

Figure S7
**PDE simulation results for Parameter Set 2 in single cell with diffusion.** Here 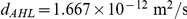. Perturbation in 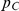 of twice the steady state value. Perturbation causes growing oscillations until stable limit cycle is reached.(TIFF)

Figure S8
**PDE simulation results for Parameter Set 2 in single cell without diffusion.** Here 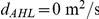. Perturbation in 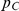 of twice the steady state value. Perturbation causes decaying oscillations, which asymptotically approach the steady state.(TIFF)

Figure S9
**Stochastic simulation results for Parameter Set 2 in single cell with diffusion.** Here 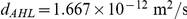. Perturbation in 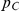 of twice the steady state value rounded to nearest molecule. Stochasticity causes growing oscillations that eventually exhibit relatively stable period and amplitude.(TIFF)

Figure S10
**Stochastic simulation results for Parameter Set 2 in single cell without diffusion.** Here 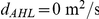. Perturbation in 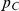 of twice the steady state value rounded to nearest molecule. Stochasticity causes sustained oscillations of short period and small amplitude. Occasional “firing events” eventually settle.(TIFF)

Figure S11
**Stochastic simulation results for Parameter Set 2 in line of cells with diffusion.** Here 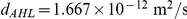. Concentrations (colorbar) given in molecules per cell. All species set to steady state values rounded to nearest molecule. Stochasticity causes growing oscillations that eventually exhibit patterning. First five of the ten species are shown here. See Figure S12 for the rest.(TIFF)

Figure S12
**Stochastic simulation results for Parameter Set 2 in line of cells with diffusion.** Here 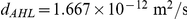. Concentrations (colorbar) given in molecules per cell. All species set to steady state values rounded to nearest molecule. Stochasticity causes growing oscillations that eventually exhibit patterning. Last five of the ten species are shown here. See Figure S11 for the rest.(TIFF)

Table S1 **Acceptable ranges and chosen parameter values for PDE and stochastic simulations.**
(PDF)

Table S2 **Steady-state concentrations given by the analysis for the parameter sets in Table S1.**
(PDF)

Table S3 **Measurements of instability for spatial waves given by the analysis for the parameter sets in Table S1.**
(PDF)

Text S1 **Analysis of quenched oscillator system for satisfying the three conditions for Turing instability.**
(PDF)

Text S2 **Bifurcation Analysis.**
(PDF)

Text S3 **Choosing parameter values for Parameter Set 1.**
(PDF)

Text S4 **Reaction set for stochastic simulations.**
(PDF)

Text S5 **Choosing parameter values for Parameter Set 2.**
(PDF)

## References

[1] Wolpert L, Beddington R, Jessell T, Lawrence P, Meyerowitz E (2002). Principles of Devel- opment. 2nd edition.

[2] Wolpert L (1969). Positional information and the spatial pattern of cellular differentiation.. J Theor Biol.

[3] Braybrook S, Kuhlemeier C (2010). How a plant builds leaves.. Plant Cell.

[4] Lewis J (2008). From signals to patterns: Space, time, and mathematics in developmental biology.. Science.

[5] Meinhardt H, Gierer A (2000). Pattern formation by local self-activation and lateral inhibition.. Bioessays.

[6] Meinhardt H (1982). Models of Biological Pattern Formation. volume 6.

[7] Murray J (2003). Mathematical Biology II: Spatial Models and Biomedical Applications. 3rd edition.

[8] Turing A (1952). The Chemical Basis of Morphogenesis.. Philos Trans R Soc London, Ser B.

[9] Cohen D, Morfino R, Maharbiz M (2009). A Modified Consumer Inkjet for Spatiotemporal Control of Gene Expression.. PLoS ONE.

[10] Sohka T, Heins R, Ostermeier M (2009). Morphogen-defined patterning of Escherichia coli enabled by an externally tunable band-pass filter.. J Biol Eng.

[11] Basu S, Gerchman Y, Collins C, Arnold F, Weiss R (2005). A synthetic multicellular system for programmed pattern formation.. Nature.

[12] Basu S, Mehreja R, Thiberge S, Chen M, Weiss R (2004). Spatiotemporal control of gene expression with pulse-generating networks.. Proc Natl Acad Sci U S A.

[13] Lucchetta E, Lee J, Fu L, Patel N, Ismagilov R (2005). Dynamics of Drosophila embryonic pattern- ing network perturbed in space and time using microfluidics.. Nature.

[14] Gierer A, Meinhardt H (1972). A theory of biological pattern formation.. Biol Cybern.

[15] Meinhardt H (2008). Models of biological pattern formation: from elementary steps to the organi- zation of embryonic axes.. Curr Top Dev Biol.

[16] Strier D, Dawson S (2004). Role of complexing agents in the appearance of Turing patterns.. Phys Rev E.

[17] Dillon R, Maini P, Othmer H (1994). Pattern formation in generalized Turing systems.. J Math Biol.

[18] Elowitz M, Leibler S (2000). A synthetic oscillatory network of transcriptional regulators.. Nature.

[19] Atkinson M, Savageau M, Myers J, Ninfa A (2003). Development of genetic circuitry exhibiting toggle switch or oscillatory behavior in escherichia coli.. Cell.

[20] Stricker J, Cookson S, Bennett M, Mather W, Tsimring L (2008). A fast, robust and tunable synthetic gene oscillator.. Nature.

[21] Winfree A (2001). The Geometry of Biological Time. 2nd edition.

[22] Garcia-Ojalvo J, Elowitz M, Strogatz S (2004). Modeling a synthetic multicellular clock: Repressi- lators coupled by quorum sensing.. Proc Natl Acad Sci U S A.

[23] Danino T, Mondragόn-Palomino O, Tsimring L, Hasty J (2010). A synchronized quorum of genetic clocks.. Nature.

[24] Kondo S, Miura T (2010). Reaction-diffusion model as a framework for understanding biological pattern formation.. Science.

[25] Meinhardt H (2004). Out-of-phase oscillations and traveling waves with unusual properties: the use of three-component systems in biology.. Physica D.

[26] Liu R, Liaw S, Maini P (2007). Oscillatory turing patterns in a simple reaction-diffusion system.. J Korean Phys Soc.

[27] Haberman R (1998). Elementary applied partial differential equations. 3rd edition.

[28] Kubitschek H, Friske J (1986). Determination of bacterial cell volume with the Coulter Counter.. J Bacteriol.

[29] Biondi E, Reisinger S, Skerker J, Arif M, Perchuk B (2006). Regulation of the bacterial cell cycle by an integrated genetic circuit.. Nature.

[30] Locke J, Kozma-Bognár L, Gould P, Fehér B, Kevei E (2006). Experimental validation of a predicted feedback loop in the multi-oscillator clock of arabidopsis thaliana.. Mol Syst Biol.

[31] Cross F (2003). Two redundant oscillatory mechanisms in the yeast cell cycle.. Cell.

[32] Kholodenko B (2006). Cell-signalling dynamics in time and space.. Nat Rev Mol Cell Biol.

[33] Lis M, Artyomov M, Devadas S, Chakraborty A (2009). Efficient stochastic simulation of reaction- diffusion processes via direct compilation.. Bioinformatics.

[34] Oppenheim A, Schafer R (2009). Discrete-Time Signal Processing. 3rd edition.

