# Supplementary figures and images for: A Feedback Quenched Oscillator Produces Turing Patterning with One Diffuser
Source: PLoS Comput Biol. 2012 Jan 26;8(1):e1002331. doi: 10.1371/journal.pcbi.1002331 (PMC3266880; doi:10.1371/journal.pcbi.1002331)

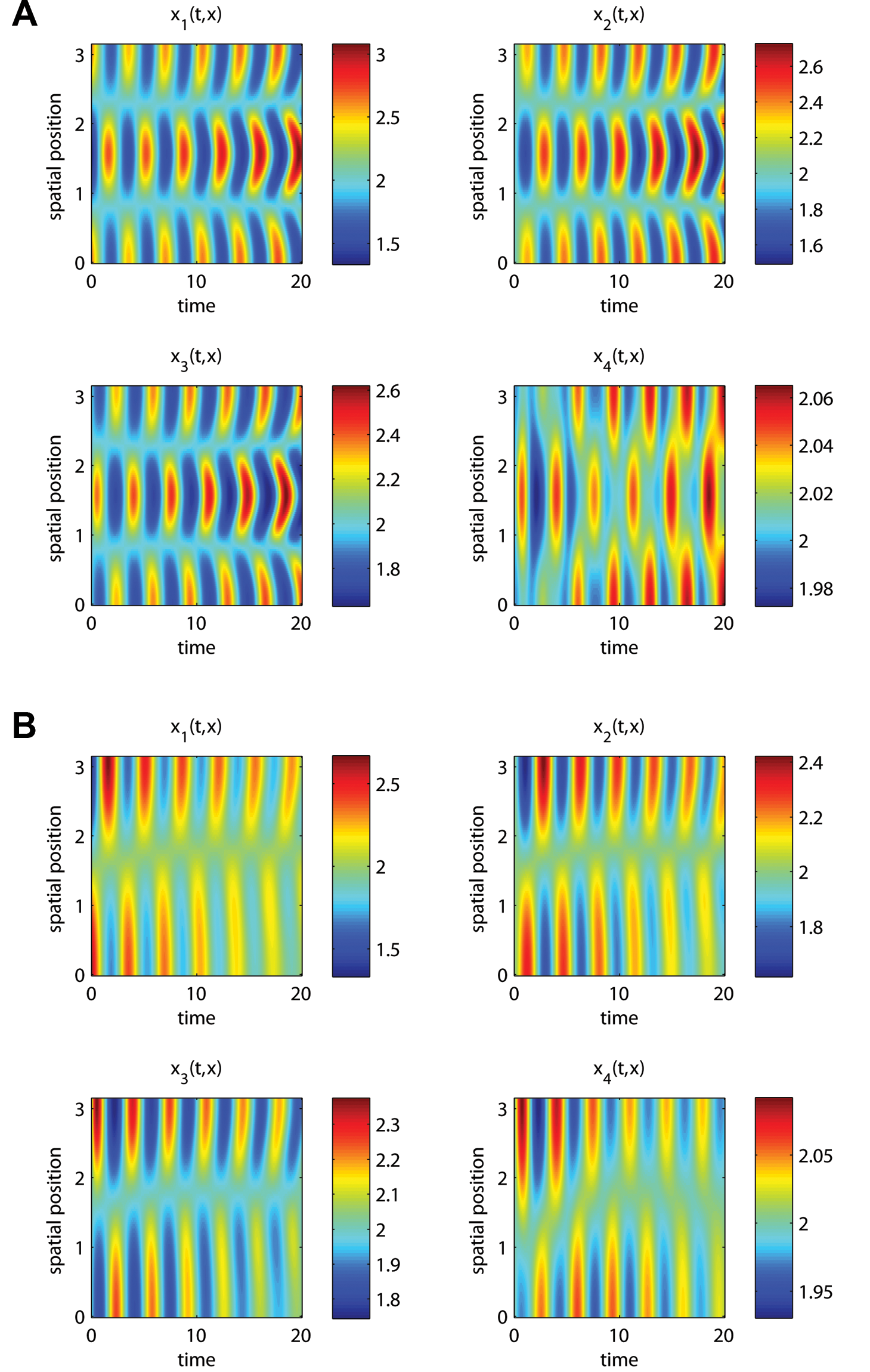

Supplement: Figure S1 — PDE simulation results for toy model in line of cells with diffusion. Here and . Position, time, and concentrations are scaled to be dimensionless. Perturbation in of amplitude steady state peak-to-peak. (A) When (wavelength ), falls above the instability threshold of and the inhomogeneity grows. (B) When (wavelength ), falls below the instability threshold of and the inhomogeneity decays. (TIFF) [file pcbi.1002331.s001.tif]

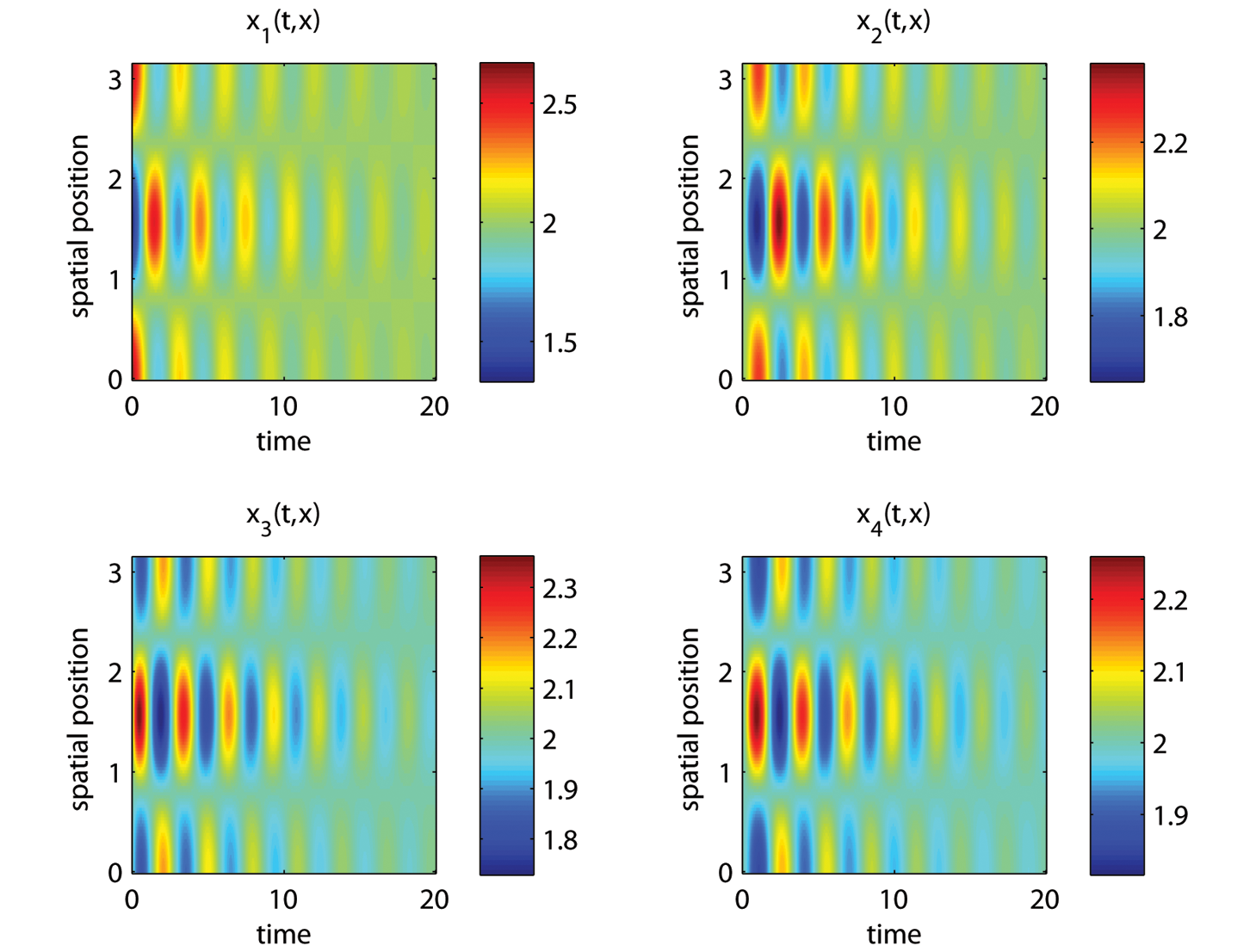

Supplement: Figure S2 — PDE simulation results for toy model in line of cells without diffusion. Here , , and (wavelength ). Position, time, and concentrations are scaled to be dimensionless. Perturbation in of amplitude steady state peak-to-peak. The cells don't communicate and each one is stable, so the inhomogeneity decays. (TIFF) [file pcbi.1002331.s002.tif]

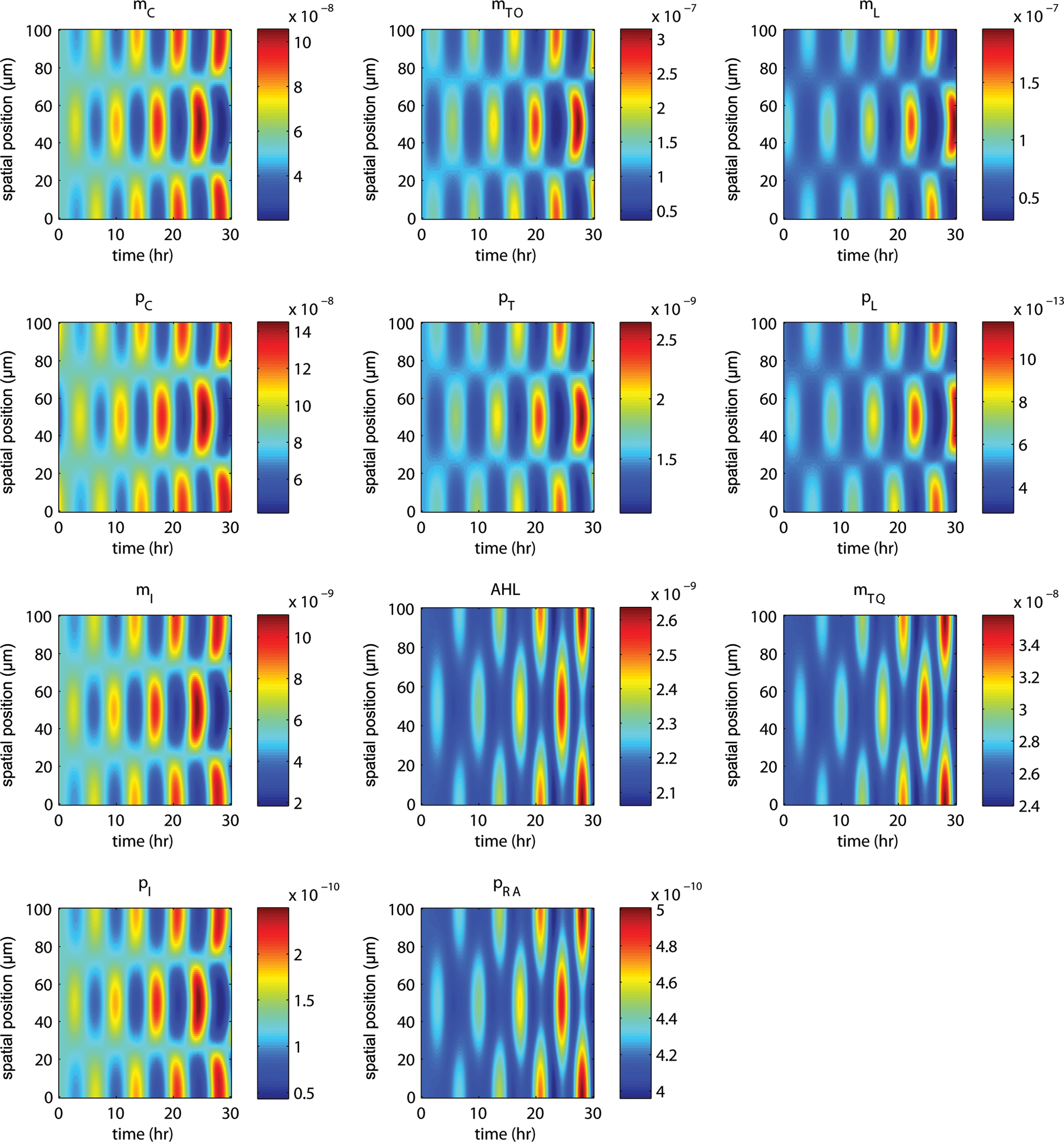

Supplement: Figure S3 — PDE simulation results for Parameter Set 1 in line of cells with diffusion and unstable wavelength. Here , , and (wavelength ). Concentrations (colorbar) given in . Perturbation in of amplitude steady state peak-to-peak. The inhomogeneity grows. (TIFF) [file pcbi.1002331.s003.tif]

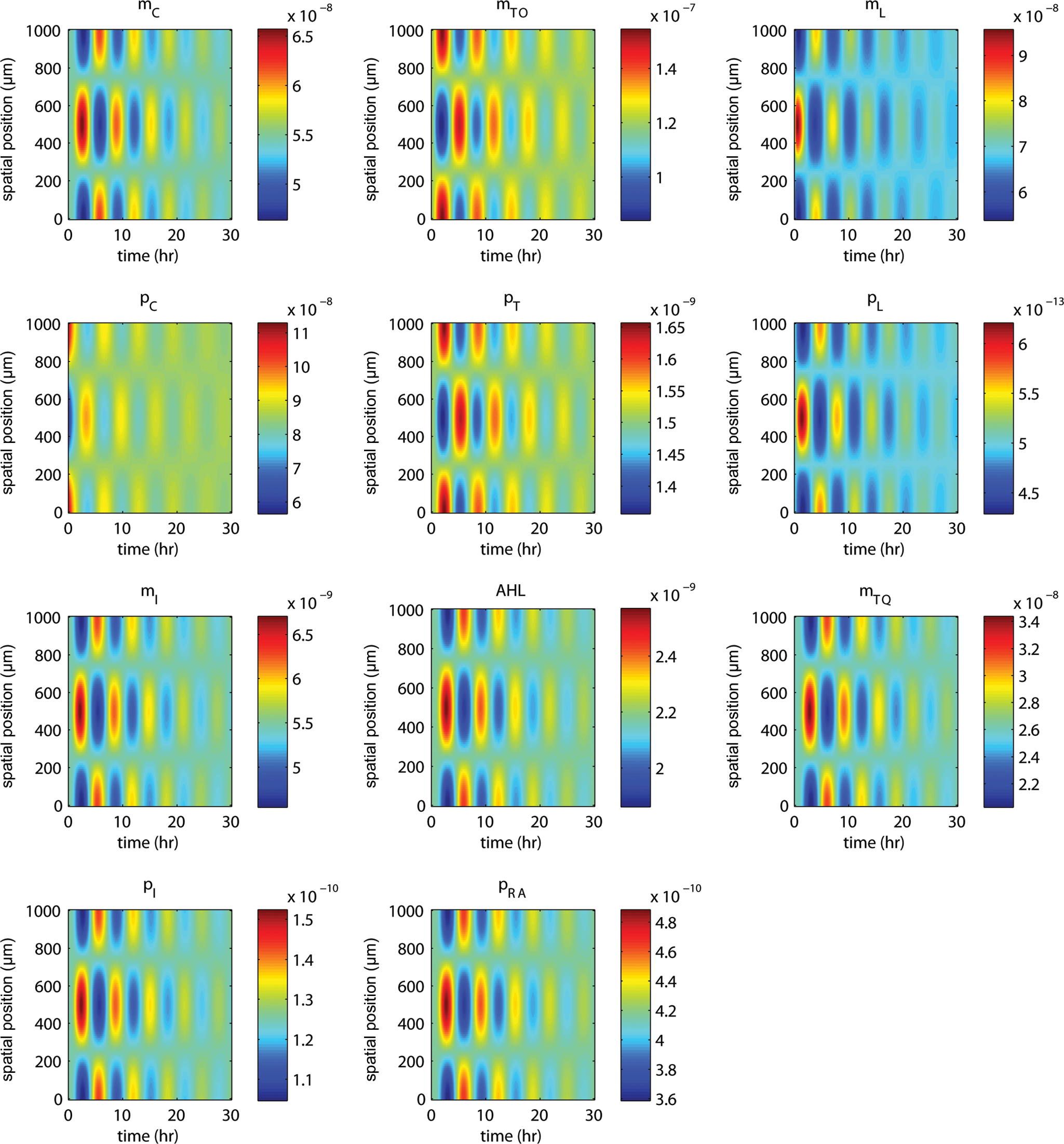

Supplement: Figure S4 — PDE simulation results for Parameter Set 1 in line of cells with diffusion and stable wavelength. Here , , and (wavelength ). Concentrations (colorbar) given in . Perturbation in of amplitude steady state peak-to-peak. The inhomogeneity decays. To achieve a stable wavelength (), we had to increase the spatial domain. (TIFF) [file pcbi.1002331.s004.tif]

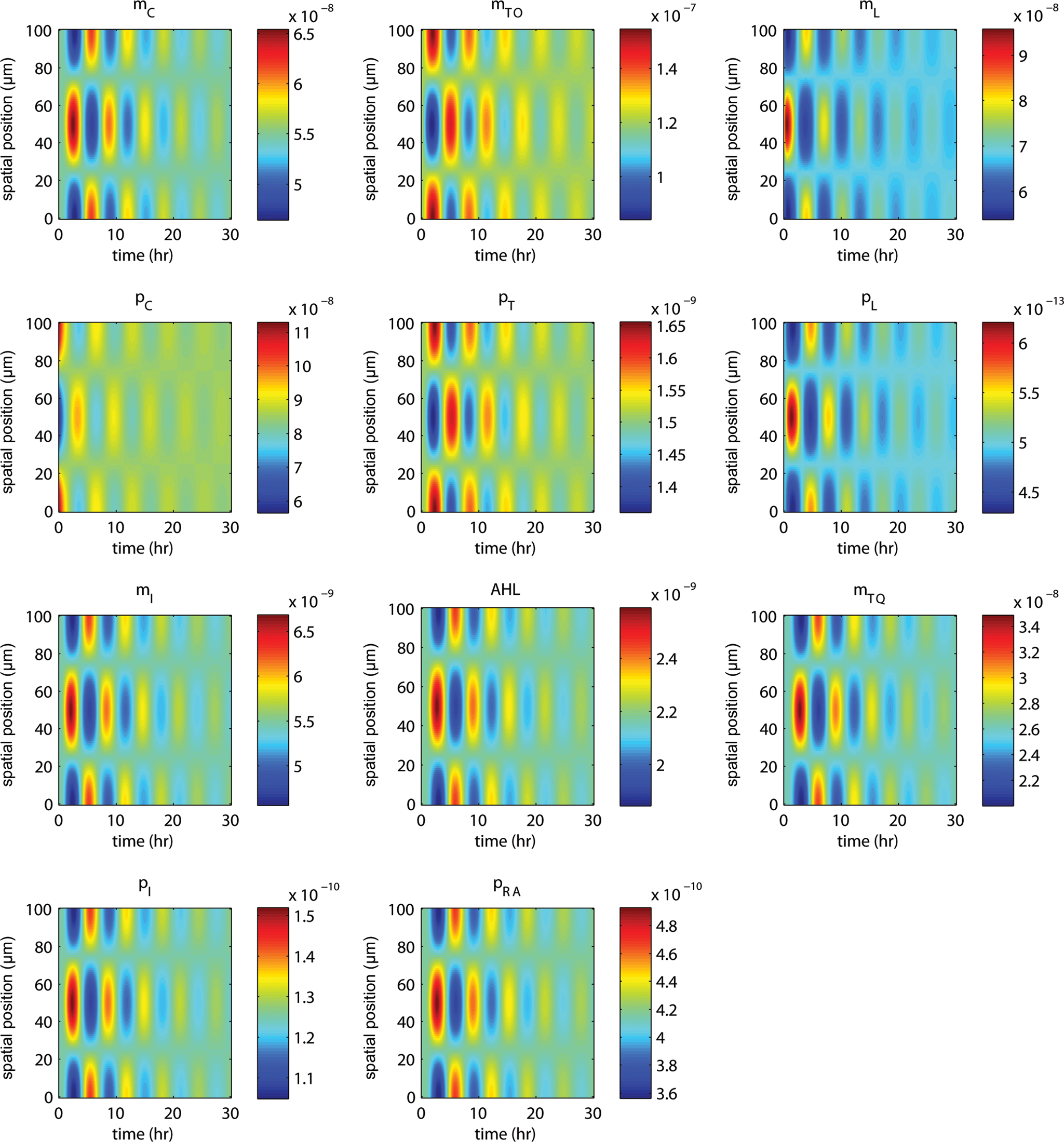

Supplement: Figure S5 — PDE simulation results for Parameter Set 1 in line of cells without diffusion. Here , , and (wavelength ). Concentrations (colorbar) given in . Perturbation in of amplitude steady state peak-to-peak. The inhomogeneity decays. (TIFF) [file pcbi.1002331.s005.tif]

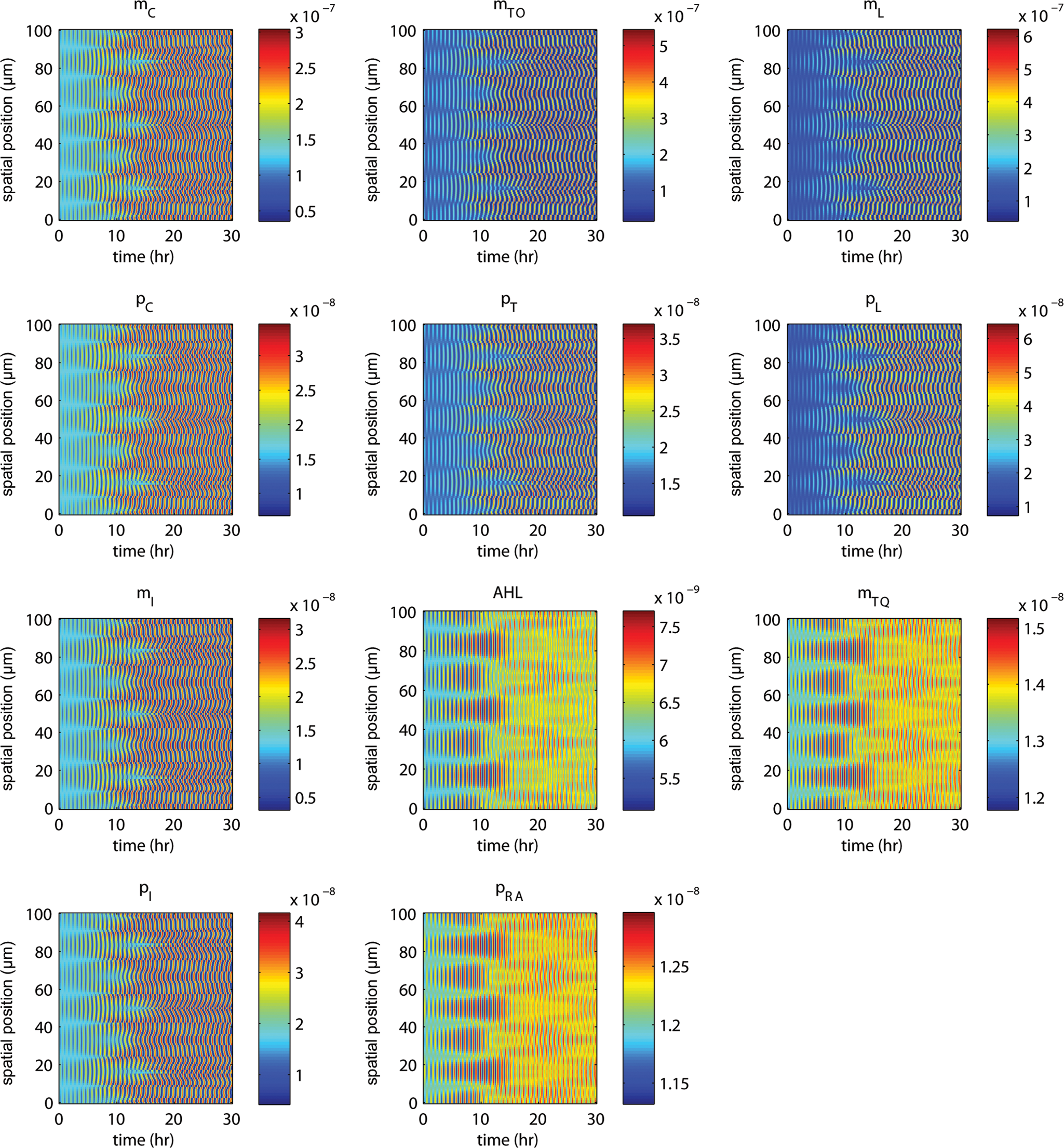

Supplement: Figure S6 — PDE simulation results for Parameter Set 2 in line of cells with diffusion. Here , , and (wavelength ). Concentrations (colorbar) given in . Perturbation in of amplitude steady state peak-to-peak. The inhomogeneity grows. (TIFF) [file pcbi.1002331.s006.tif]

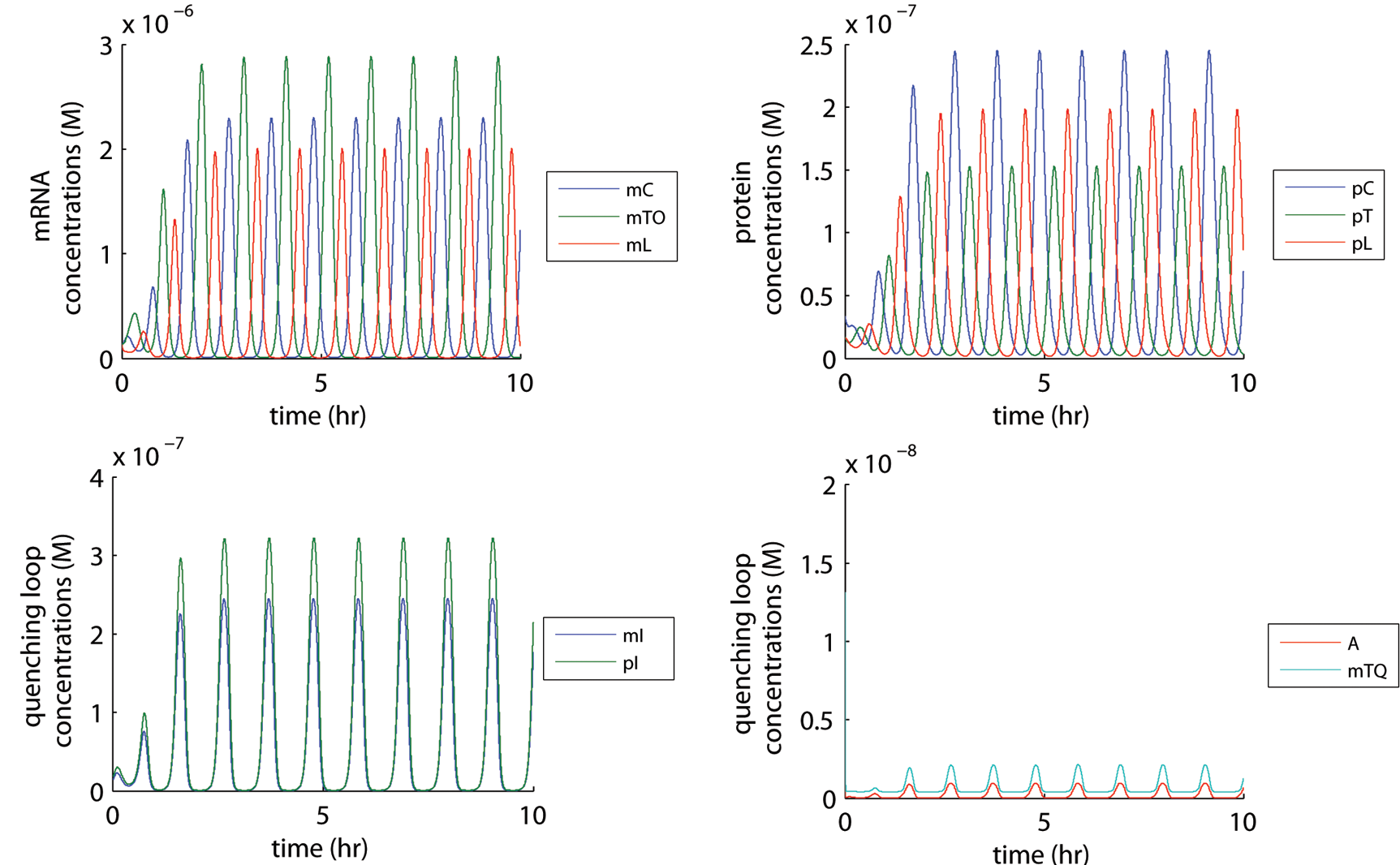

Supplement: Figure S7 — PDE simulation results for Parameter Set 2 in single cell with diffusion. Here . Perturbation in of twice the steady state value. Perturbation causes growing oscillations until stable limit cycle is reached. (TIFF) [file pcbi.1002331.s007.tif]

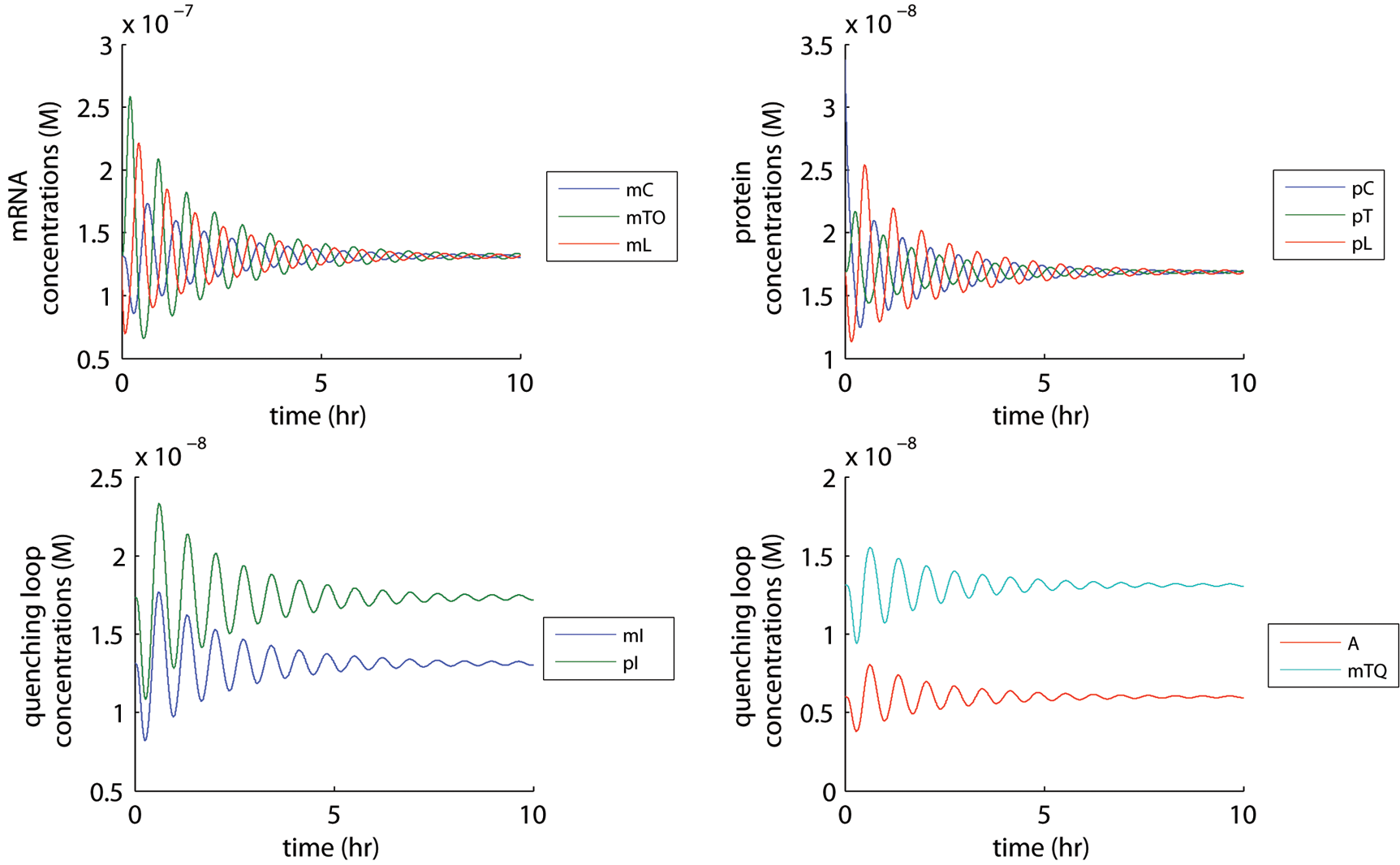

Supplement: Figure S8 — PDE simulation results for Parameter Set 2 in single cell without diffusion. Here . Perturbation in of twice the steady state value. Perturbation causes decaying oscillations, which asymptotically approach the steady state. (TIFF) [file pcbi.1002331.s008.tif]

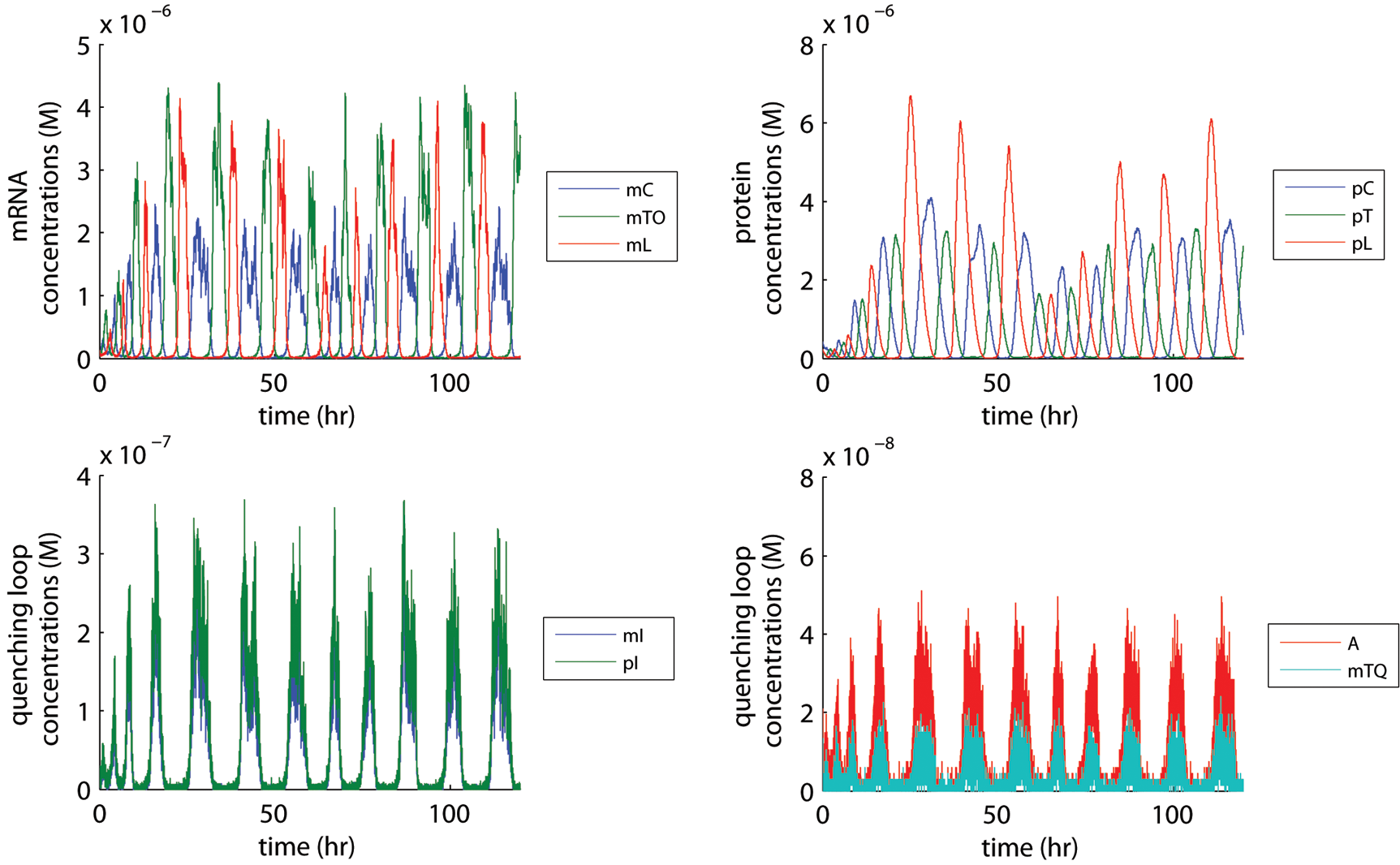

Supplement: Figure S9 — Stochastic simulation results for Parameter Set 2 in single cell with diffusion. Here . Perturbation in of twice the steady state value rounded to nearest molecule. Stochasticity causes growing oscillations that eventually exhibit relatively stable period and amplitude. (TIFF) [file pcbi.1002331.s009.tif]

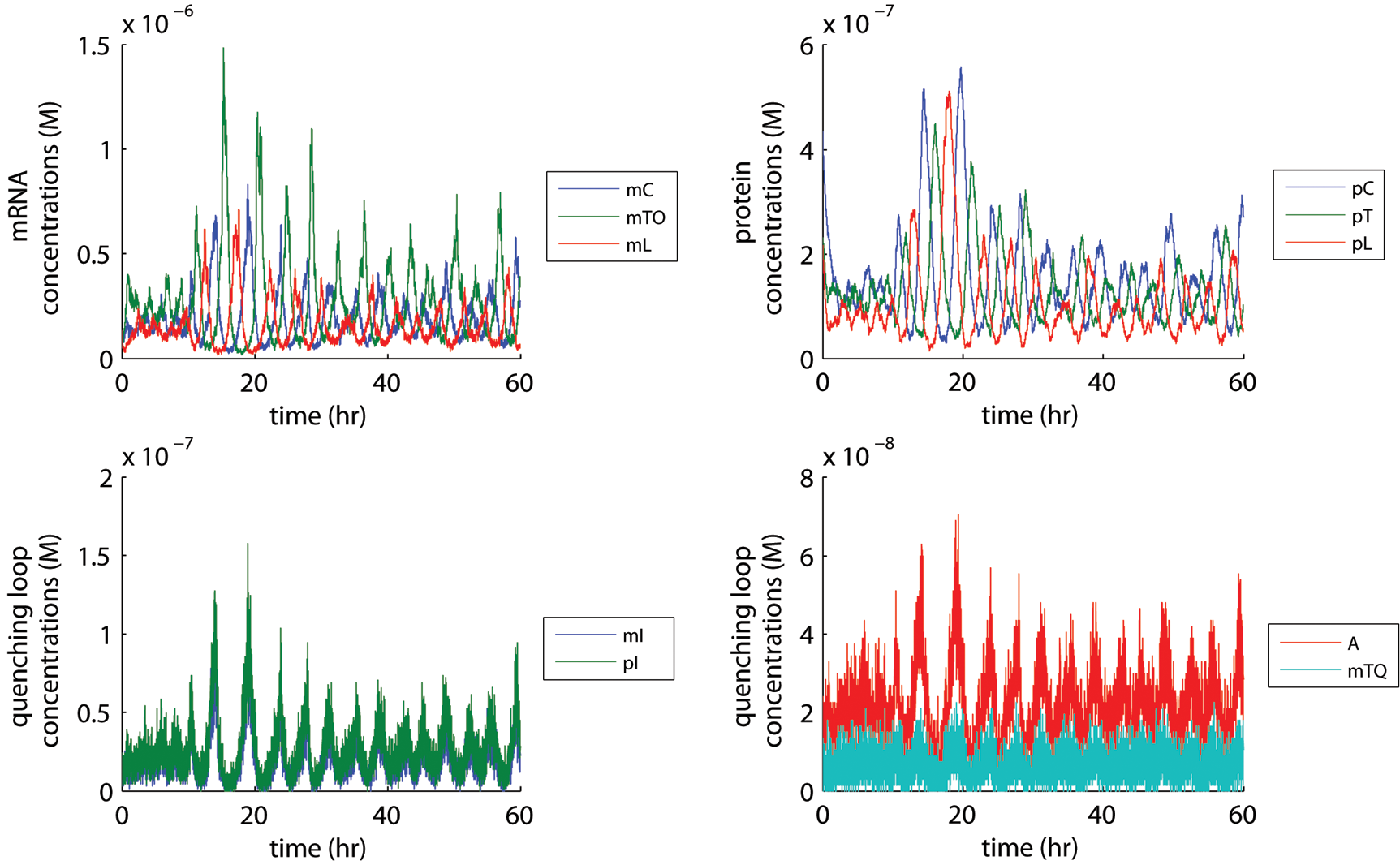

Supplement: Figure S10 — Stochastic simulation results for Parameter Set 2 in single cell without diffusion. Here . Perturbation in of twice the steady state value rounded to nearest molecule. Stochasticity causes sustained oscillations of short period and small amplitude. Occasional “firing events” eventually settle. (TIFF) [file pcbi.1002331.s010.tif]

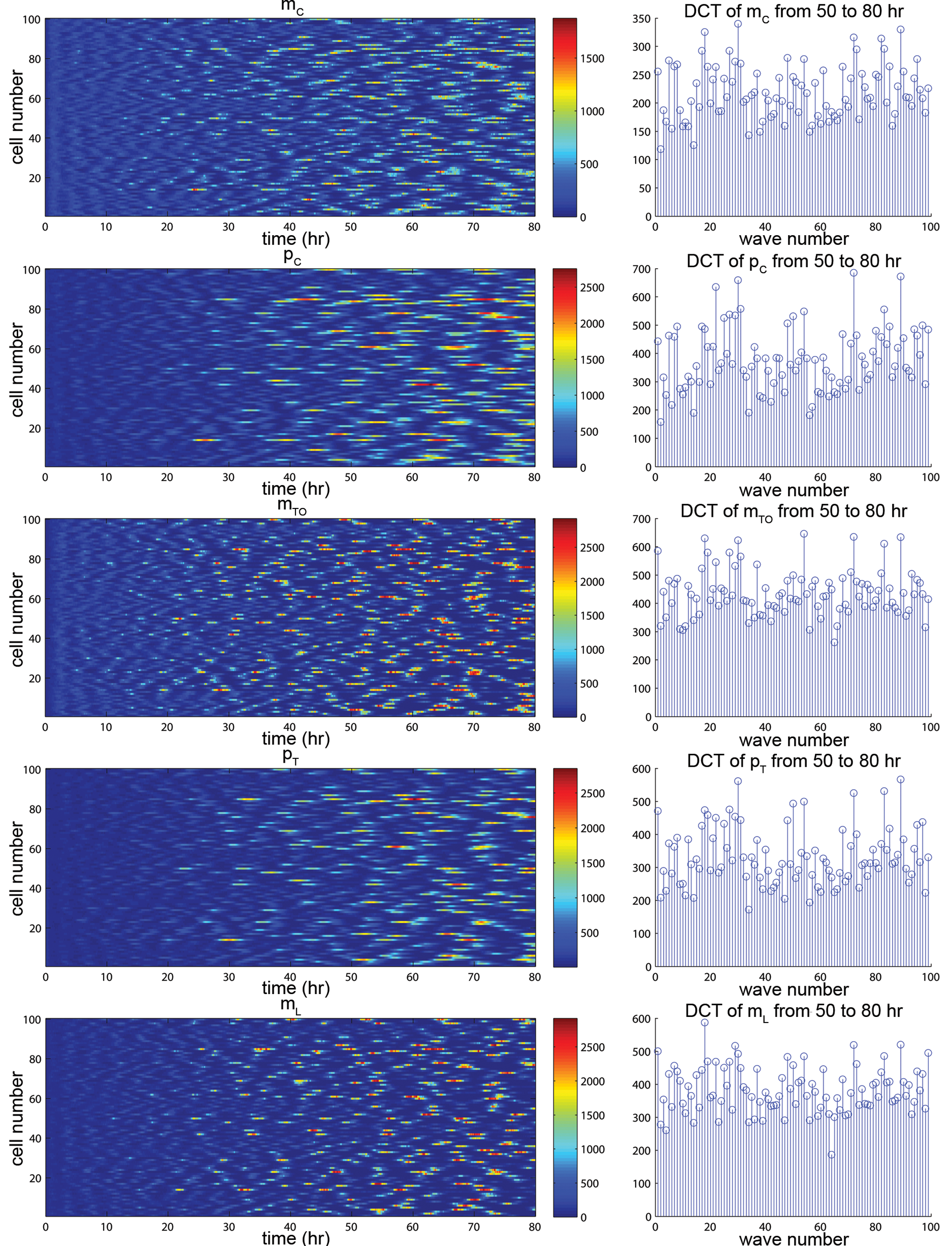

Supplement: Figure S11 — Stochastic simulation results for Parameter Set 2 in line of cells with diffusion. Here . Concentrations (colorbar) given in molecules per cell. All species set to steady state values rounded to nearest molecule. Stochasticity causes growing oscillations that eventually exhibit patterning. First five of the ten species are shown here. See Figure S12 for the rest. (TIFF) [file pcbi.1002331.s011.tif]

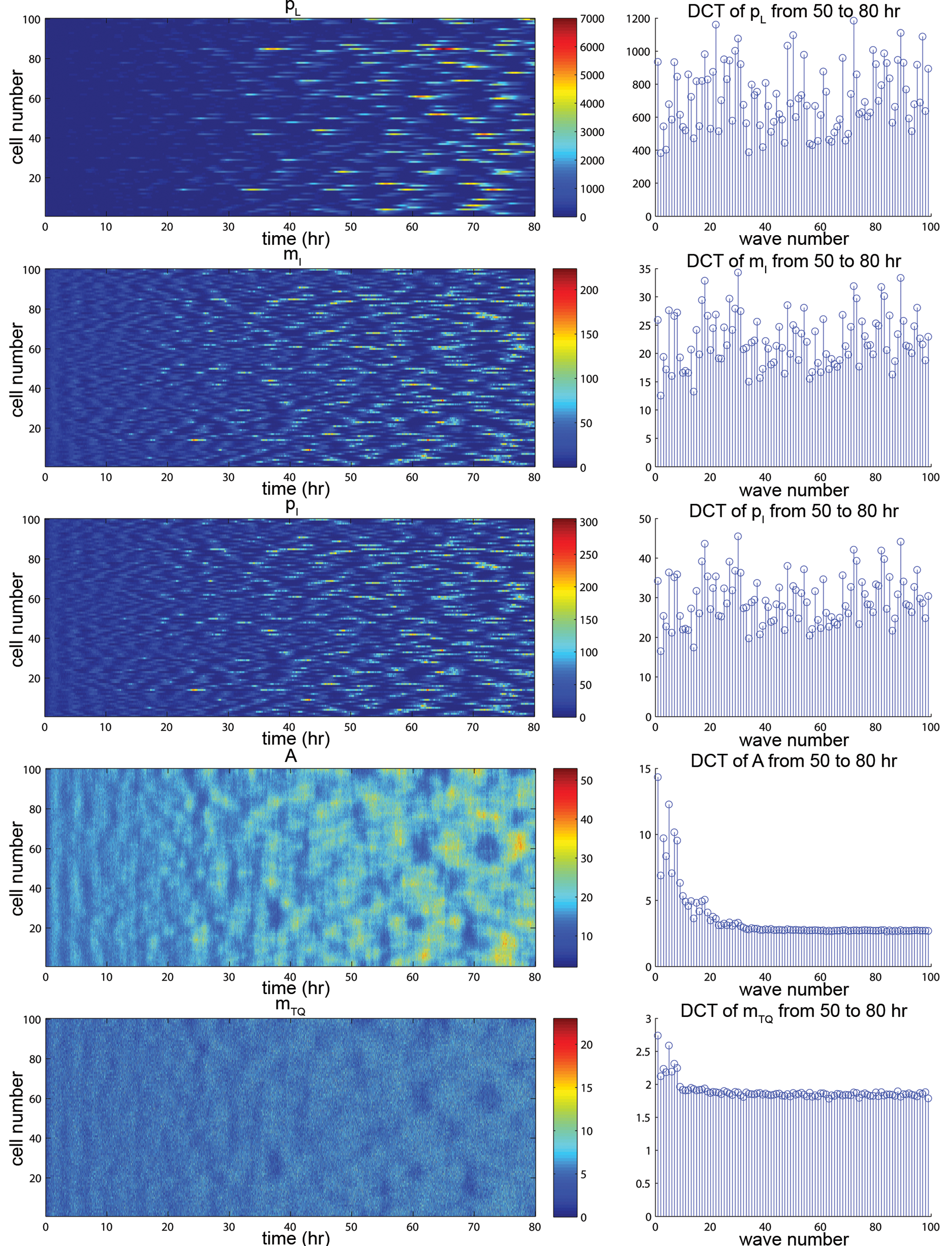

Supplement: Figure S12 — Stochastic simulation results for Parameter Set 2 in line of cells with diffusion. Here . Concentrations (colorbar) given in molecules per cell. All species set to steady state values rounded to nearest molecule. Stochasticity causes growing oscillations that eventually exhibit patterning. Last five of the ten species are shown here. See Figure S11 for the rest. (TIFF) [file pcbi.1002331.s012.tif]
