# Supplementary material for: A Feedback Quenched Oscillator Produces Turing Patterning with One Diffuser
Source: PLoS Comput Biol. 2012 Jan 26;8(1):e1002331. doi: 10.1371/journal.pcbi.1002331 (PMC3266880; doi:10.1371/journal.pcbi.1002331)
Supplement: Table S1 — Acceptable ranges and chosen parameter values for PDE and stochastic simulations. (PDF) [file pcbi.1002331.s013.pdf]

Table S1: Acceptable ranges and chosen parameter values for PDE and stochastic simulations.

**Parameter**	**Range**	**Value for PDE Simulation** (Parameter Set 1)	**Value for Stochastic Simulation** (Parameter Set 2)
γ_C	$\gamma_x \leq 1 \times 10^{-2}$ s^{-1} [s12]	2.89×10^{-4} s^{-1}	2.89×10^{-3} s^{-1}
γ_T		2.89×10^{-4} s^{-1}	2.89×10^{-3} s^{-1}
γ_L		2.89×10^{-4} s^{-1}	2.89×10^{-3} s^{-1}
γ_I		1.16×10^{-3} s^{-1}	2.89×10^{-2} s^{-1}
γ_A	$\gamma_A \sim 2 \times 10^{-3}$ s^{-1} [s13]	7.70×10^{-4} s^{-1}	2.89×10^{-2} s^{-1}
γ_{mO}	$3.5 \times 10^{-4} \leq \gamma_m \leq 2.3 \times 10^{-2}$ s^{-1} [s14]	5.78×10^{-4} s^{-1}	5.78×10^{-3} s^{-1}
γ_{mQ}		5.78×10^{-3} s^{-1}	5.78×10^{-2} s^{-1}
$V_{P_{LtetO-1}}$	$V_x \leq 4$ s^{-1} [s15]	0.3s^{-1} [s16]	4 s^{-1}
$V_{P_{LlacO-1}}$		0.23 s^{-1} [s16]	4 s^{-1}
V_{P_R}		0.06 s^{-1} [s17]	4 s^{-1}
$V_{P_{LuxI}}$		0.26 s^{-1} [s18]	4 s^{-1}
N_C	$1 \leq N_x \leq 30$	5	4
N_{TO}		5	4
N_L		5	4
N_I		5	4
N_{TQ}		5	4
C		1.5×10^{-9} M [s19]	1.5×10^{-9} M
K_C	$1 \times 10^{-13} \leq K_x \leq 1 \times 10^{-7}$ M [s20, 21]	2.5×10^{-8} M [s21]	3×10^{-9} M
K_T		1.786×10^{-10} M [s22]	3×10^{-9} M
K_L		1×10^{-13} M [s20]	3×10^{-9} M
K_{RA}		1.5×10^{-9} M [s18]	6.75×10^{-9} M
n_C		2 [s21, 23]	2
n_T		2 [s23]	2
n_L		2 [s23]	2
n_{RA}		2 [s18]	2
$\ell_{P_{LtetO-1}}$		$1/5050$ [s16]	$1/1000$
$\ell_{P_{LlacO-1}}$		$1/620$ [s16]	$1/1000$
ℓ_{P_R}		$1/131$ [s24]	$1/1000$
$\ell_{P_{LuxI}}$		$1/167$ [s18]	$1/1000$
ϵ_C	$\epsilon_x \leq 5.78 \times 10^{-3}$ s^{-1} [s25]	4.470×10^{-4} s^{-1}	3.712×10^{-4} s^{-1}
ϵ_{TO}		2.269×10^{-6} s^{-1}	1.856×10^{-4} s^{-1}
ϵ_L		2.113×10^{-9} s^{-1}	3.712×10^{-4} s^{-1}
ϵ_I		2.655×10^{-5} s^{-1}	3.815×10^{-2} s^{-1}
ϵ_{TQ}		6.224×10^{-6} s^{-1}	1.856×10^{-3} s^{-1}
v_3		0.01335 s^{-1} [s26]	0.01 s^{-1}
k_f	$1 \times 10^4 \leq k_f \leq 5 \times 10^9$ M [s27]	1×10^9 M^{-1}s^{-1} [s27]	5×10^9 M^{-1}s^{-1}
k_r	$k_r \geq 5 \times 10^{-4}$ s^{-1} (est. from [s18, 27, 28])	50 s^{-1} (est. from [s18, 27, 28])	15 s^{-1}
p_R		1×10^{-8} M	1.8×10^{-8} M
d_{AHL}		1.667×10^{-12} m^{2}s^{-1} (est. from [s29])	1.667×10^{-12} m^{2}s^{-1}

References

[s12] Lies M, Maurizi M (2008) Turnover of endogenous SsrA-tagged proteins mediated by ATP-dependent proteases in *Escherichia coli*. Journal of Biological Chemistry 283: 22918–22929.

[s13] Danino T, Mondragón-Palomino O, Tsimring L, Hasty J (2010) A synchronized quorum of genetic clocks. Nature 463: 326–330.

[s14] Rauhut R, Klug G (1999) mRNA degradation in bacteria. FEMS microbiology reviews 23: 353–370.

[s15] Jia Y, Patel S (1997) Kinetic mechanism of transcription initiation by bacteriophage T7 RNA polymerase. Biochemistry 36: 4223–4232.

[s16] Lutz R, Bujard H (1997) Independent and tight regulation of transcriptional units in Escherichia coli via the LacR/O, the TetR/O and AraC/I_2-I_2 regulatory elements. Nucleic acids research 25: 1203.

[s17] Potrykus K, Wegrzyn G, Hernandez V (2002) Multiple Mechanisms of Transcription Inhibition by ppGpp at the λp_R Promoter. Journal of Biological Chemistry 277: 43785–43791.

[s18] Canton B, Labno A, Endy D (2008) Refinement and standardization of synthetic biological parts and devices. Nature biotechnology 26: 787–793.

[s19] Kubitschek H, Friske J (1986) Determination of bacterial cell volume with the Coulter Counter. Journal of Bacteriology 168: 1466-1467.

[s20] Riggs A, Suzuki H, Bourgeois S (1970) lac repressor-operator interaction: I. Equilibrium studies. Journal of Molecular Biology 48: 67–83.

[s21] Hawley D, Johnson A, McClure W (1985) Functional and physical characterization of transcription initiation complexes in the bacteriophage lambda OR region. Journal of Biological Chemistry 260: 8618–8626.

[s22] Meier I, Wray L, Hillen W (1988) Differential regulation of the Tn10-encoded tetracycline resistance genes tetA and tetR by the tandem tet operators O1 and O2. The EMBO journal 7: 567–572.

[s23] Bintu L, Buchler N, Garcia H, Gerland U, Hwa T, et al. (2005) Transcriptional regulation by the numbers: applications. Current opinion in genetics & development 15: 125–135.

[s24] Dodd I, Shearwin K, Perkins A, Burr T, Hochschild A, et al. (2004) Cooperativity in long-range gene regulation by the λ CI repressor. Genes & development 18: 344–354.

[s25] Kierzek A, Zaim J, Zielenkiewicz P (2001) The effect of transcription and translation initiation frequencies on the stochastic fluctuations in prokaryotic gene expression. Journal of Biological Chemistry 276: 8165–8172.

[s26] Schaefer A, Val D, Hanzelka B, Cronan J, Greenberg E (1996) Generation of cell-to-cell signals in quorum sensing: acyl homoserine lactone synthase activity of a purified Vibrio fischeri LuxI protein. Proceedings of the National Academy of Sciences of the United States of America 93: 9505–9509.

[s27] Zhou H (1997) Enhancement of protein-protein association rate by interaction potential: accuracy of prediction based on local Boltzmann factor. Biophysical journal 73: 2441–2445.

[s28] Adar Y, Ulitzur S (1993) GroESL proteins facilitate binding of externally added inducer by LuxR protein-containing E. coli cells. Journal of bioluminescence and chemiluminescence 8: 261–266.

[s29] Basu S, Gerchman Y, Collins C, Arnold F, Weiss R (2005) A synthetic multicellular system for programmed pattern formation. Nature 434: 1130–1134.
