# Supplementary material for: A Feedback Quenched Oscillator Produces Turing Patterning with One Diffuser
Source: PLoS Comput Biol. 2012 Jan 26;8(1):e1002331. doi: 10.1371/journal.pcbi.1002331 (PMC3266880; doi:10.1371/journal.pcbi.1002331)
Supplement: Table S2 — Steady-state concentrations given by the analysis for the parameter sets in Table S1. (PDF) [file pcbi.1002331.s014.pdf]

Table S2: Steady-state concentrations given by the analysis for the parameter sets in Table S1.

**Species**	**Steady-state Concentration for PDE Simulation** (Parameter Set 1)	**Steady-state Concentration for Stochastic Simulation** (Parameter Set 2)
m_C	5.478×10^{-8} M	1.314×10^{-7} M
p_C	8.474×10^{-8} M	1.687×10^{-8} M
m_{TO}	1.200×10^{-7} M	1.314×10^{-7} M
p_T	1.506×10^{-9} M	1.687×10^{-8} M
m_L	6.828×10^{-8} M	1.314×10^{-7} M
p_L	4.992×10^{-13} M	1.687×10^{-8} M
m_I	5.478×10^{-9} M	1.314×10^{-8} M
p_I	1.254×10^{-11} M	1.734×10^{-8} M
A	2.174×10^{-9} M	6.000×10^{-9} M
p_{RA}	4.166×10^{-10} M	1.200×10^{-8} M
m_{TQ}	2.619×10^{-8} M	1.314×10^{-8} M
