# Supplementary material for: A Feedback Quenched Oscillator Produces Turing Patterning with One Diffuser
Source: PLoS Comput Biol. 2012 Jan 26;8(1):e1002331. doi: 10.1371/journal.pcbi.1002331 (PMC3266880; doi:10.1371/journal.pcbi.1002331)
Supplement: Table S3 — Measurements of instability for spatial waves given by the analysis for the parameter sets in Table S1. (PDF) [file pcbi.1002331.s015.pdf]

Table S3: Measurements of instability for spatial waves given by the analysis for the parameter sets in Table S1.

**Instability Measurement**	**Value for PDE Simulation** (Parameter Set 1)	**Value for Stochastic Simulation** (Parameter Set 2)
Instability threshold d_{thresh}	9.5×10^{-4}	2.657×10^{-2}
Maximum unstable wavelength	832.3 μm	49.77 μm
Minimum unstable wave number k for $L = 100\mu m$	all are unstable	5
Minimum unstable wave number k for $L = 1000\mu m$	3	41
