# Supplementary material for: A Feedback Quenched Oscillator Produces Turing Patterning with One Diffuser
Source: PLoS Comput Biol. 2012 Jan 26;8(1):e1002331. doi: 10.1371/journal.pcbi.1002331 (PMC3266880; doi:10.1371/journal.pcbi.1002331)
Supplement: Text S1 — Analysis of quenched oscillator system for satisfying the three conditions for Turing instability. (PDF) [file pcbi.1002331.s016.pdf]

Text S1 Analysis of quenched oscillator system for satisfying the three conditions for Turing instability

To obtain parameter constraints for Turing instability, we first linearize the reaction terms in (6)-(16) at the steady state $(\bar{m}_C, \bar{p}_C, \bar{m}_{TO}, \bar{p}_T, \bar{m}_L, \bar{p}_L, \bar{m}_I, \bar{p}_I, \bar{A}, \bar{p}_{RA}, \bar{m}_{TQ})$ and obtain the Jacobian matrix:

$$J = \left[\begin{array}{cccccc|ccccc} -\gamma_{mO} & 0 & 0 & -b_4 & 0 & 0 & 0 & 0 & 0 & 0 & 0 \\ \epsilon_C & -\gamma_C & 0 & 0 & 0 & 0 & 0 & 0 & 0 & 0 & 0 \\ 0 & 0 & -\gamma_{mO} & 0 & 0 & -b_6 & 0 & 0 & 0 & 0 & 0 \\ 0 & 0 & \epsilon_{TO} & -\gamma_T & 0 & 0 & 0 & 0 & 0 & 0 & \epsilon_{TQ} \\ 0 & -b_2 & 0 & 0 & -\gamma_{mO} & 0 & 0 & 0 & 0 & 0 & 0 \\ 0 & 0 & 0 & 0 & \epsilon_L & -\gamma_L & 0 & 0 & 0 & 0 & 0 \\ \hline 0 & 0 & 0 & -b_{42} & 0 & 0 & -\gamma_{mQ} & 0 & 0 & 0 & 0 \\ 0 & 0 & 0 & 0 & 0 & 0 & \epsilon_I & -\gamma_I & 0 & 0 & 0 \\ 0 & 0 & 0 & 0 & 0 & 0 & 0 & v_3 & -a_9 & a_{10} & 0 \\ 0 & 0 & 0 & 0 & 0 & 0 & 0 & 0 & c_9 & -a_{10} & 0 \\ 0 & 0 & 0 & 0 & 0 & 0 & 0 & 0 & 0 & b_{10} & -\gamma_{mQ} \end{array}\right],$$

where we use the parameters $\alpha_C = \bar{p}_C/K_C$, $\alpha_T = \bar{p}_T/K_T$, $\alpha_L = \bar{p}_L/K_L$, and $\alpha_A = \bar{A}/K_A$ to obtain the off-diagonal entries:

$$\begin{aligned} c_9 &= \tfrac{k_f p_R}{1+\alpha_A}, \\ a_9 &= c_9 + \gamma_A, \\ a_{10} &= k_r(1+\alpha_A), \\ b_2 &= V_{P_R} V_L C \tfrac{n_C \alpha_C^{(n_C-1)}}{K_C(1+\alpha_C^{n_C})^2}, \\ b_4 &= V_{P_{LtetO-1}} N_C C \tfrac{n_T \alpha_T^{(n_T-1)}}{K_T(1+\alpha_T^{n_T})^2}, \\ b_{42} &= V_{P_{LtetO-1}} N_I C \tfrac{n_T \alpha_T^{(n_T-1)}}{K_T(1+\alpha_T^{n_T})^2}, \\ b_6 &= V_{P_{LlacO-1}} N_{TO} C \tfrac{n_L \alpha_L^{(n_L-1)}}{K_L(1+\alpha_L^{n_L})^2}, \\ b_{10} &= V_{P_{LuxI}} N_{TQ} C \tfrac{n_{RA}(\frac{K_{RA}}{p_R})(\frac{K_{RA}}{p_R}\frac{1+\alpha_A}{\alpha_A})^{n_{RA}}}{p_R(1+(\frac{K_{RA}}{p_R}\frac{1+\alpha_A}{\alpha_A})^{n_{RA}})^2}. \end{aligned}$$

J_{osc} is the 6×6 principal submatrix of J, which corresponds to the first loop – the standard repressilator system (*cI-lacI-tetR*). AHL is the only diffusible species, so $D = \text{diag}\{0, 0, 0, 0, 0, 0, 0, 0, d_{AHL}, 0, 0\}$.

Condition 1: The oscillator loop by itself would produce oscillations (J_{osc} is unstable).

The eigenvalues of J_{osc} are the roots of:

$$\det(\lambda I - J_{osc}) = (\lambda + \gamma_{mO})^3 (\lambda + \gamma_p)^3 + \epsilon_C \epsilon_{TO} \epsilon_L b_2 b_4 b_6. \tag{S.1}$$

It can be shown [s9] that instability of J_{osc} is achieved when:

$$\frac{(\beta+1)^2}{\beta} < \frac{3X^2}{4+2X} \tag{S.2}$$

where $\beta = \gamma_p/\gamma_{mO}$ and $X = -\frac{1}{\gamma_p \gamma_{mO}} \sqrt[3]{\epsilon_C \epsilon_{TO} \epsilon_L b_2 b_4 b_6}$. Substituting steady-state expressions and rearranging, we arrive at the following expression for X:

$$\begin{aligned} X^3 \;=\; & -n_C n_T n_L \frac{\alpha_{RA}}{1+\alpha_{RA}} \frac{\alpha_C^{n_C}}{1+\alpha_C^{n_C}} \frac{1}{1+\ell_{P_R}(1+\alpha_C^{n_C})} \frac{\alpha_T^{n_T}}{1+\alpha_T^{n_T}} \frac{1}{1+\ell_{P_{LtetO-1}}(1+\alpha_T^{n_T})} \\ & \times \frac{\alpha_L^{n_L}}{1+\alpha_L^{n_L}} \frac{1}{1+\ell_{P_{LlacO-1}}(1+\alpha_L^{n_L})}, \end{aligned} \tag{S.3}$$

where the additional variable $\alpha_{RA} \geq 0$ is defined by the relation:

$$\frac{1}{1+\alpha_{RA}} = \frac{\epsilon_{TQ} V_{P_{LuxI}} N_{TQ} C}{\gamma_p \gamma_{mQ} \alpha_T K_T} \left(\frac{1}{1+(\frac{K_{RA}}{p_R}\frac{1+\alpha_A}{\alpha_A})^{n_{RA}}} + \ell_{P_{LuxI}} \right).$$

Condition 2: The quenching loop ceases oscillations in the full system (J is stable).

The eigenvalues of J are the roots of:

$$\det(\lambda I - J) = \det(\lambda I - J_{osc})(\lambda + \gamma_I)(\lambda + \gamma_{mQ})^2[(\lambda + a_9)(\lambda + a_{10}) - c_9 a_{10}] + F(\lambda + \gamma_{mO})^3(\lambda + \gamma_p)^2, \quad \text{(S.4)}$$

where $F = v_3 \epsilon_I \epsilon_{TQ} c_9 b_{42} b_{10}$ characterizes the feedback strength. To quench the oscillatory modes of J_{osc}, F must be a value such that all of the eigenvalues of J have negative real part. Substituting steady-state expressions and rearranging, we arrive at the following expression for F:

$$\begin{aligned} F &= \gamma_T \gamma_I \gamma_A \gamma_{mQ}^2 k_r n_T n_{RA} \frac{1}{1+\alpha_{RA}} \frac{(\frac{K_{RA}}{p_R}\frac{1+\alpha_A}{\alpha_A})^{n_{RA}}}{1+(\frac{K_{RA}}{p_R}\frac{1+\alpha_A}{\alpha_A})^{n_{RA}}} \frac{1}{1+\ell_{P_{LuxI}}(1+(\frac{K_{RA}}{p_R}\frac{1+\alpha_A}{\alpha_A})^{n_{RA}})} \\ &\quad \times \frac{\alpha_T^{n_T}}{1+\alpha_T^{n_T}} \frac{1}{1+\ell_{P_{LtetO-1}}(1+\alpha_T^{n_T})}. \end{aligned} \quad \text{(S.5)}$$

Condition 3: Diffusion will weaken the quenching loop's influence on the oscillator loop for high wave numbers, allowing spatio-temporal oscillations to emerge ($J + \lambda_k D$ is unstable for some $k \geq 1$).

For $\Omega = [0, L]$, $\lambda_k = -(k\pi/L)^2$ for eigenfunctions $\cos(\frac{k\pi}{L}x)$. $J + \lambda_k D$ looks identical to J except for the AHL entry of the diagonal, which is now defined as $-\hat{a}_9 = -c_9 - \gamma_A + \lambda_k d_{AHL}$. This leads to:

$$\det(\lambda I - (J + \lambda_k D)) = \det(\lambda I - J_{osc})(\lambda + \gamma_I)(\lambda + \gamma_{mQ})^2[(\lambda + \hat{a}_9)(\lambda + a_{10}) - c_9 a_{10}] + F(\lambda + \gamma_{mO})^3(\lambda + \gamma_p)^2, \quad \text{(S.6)}$$

which yields unstable roots for large enough $\lambda_k d_{AHL}$. This implies that, for diffusion-driven patterning, we need a large diffusion coefficient, a large wave number, or a small spatial domain. Let d_{thresh} be the instability threshold for a particular set of parameters, that is, (S.6) becomes unstable when:

$$(k\pi/L)^2 d_{AHL} > d_{thresh}.$$

This expression can be rewritten in terms of the spatial wavelength ω_x as:

$$\omega_x^2 < 4\pi^2 d_{AHL}/d_{thresh}.$$

This maximum unstable wavelength is a convenient formulation because it applies to any chosen spatial domain size.

References

[s1] Elowitz M, Leibler S (2000) A synthetic oscillatory network of transcriptional regulators. Nature 403: 335–338.
