# Supplementary material for: A Feedback Quenched Oscillator Produces Turing Patterning with One Diffuser
Source: PLoS Comput Biol. 2012 Jan 26;8(1):e1002331. doi: 10.1371/journal.pcbi.1002331 (PMC3266880; doi:10.1371/journal.pcbi.1002331)
Supplement: Text S2 — Bifurcation Analysis. (PDF) [file pcbi.1002331.s017.pdf]

Text S2 Bifurcation Analysis

Here we address and make connections to the body of literature that studies the relationship between Hopf bifurcations and Turing bifurcations [s2]. As was shown in that paper, our system is the case where a spatio-temporally oscillating solution is obtained in the presence of only a Turing bifurcation. In the bifurcation diagrams shown below, the eigenvalues of the spatial modes are shown to become unstable as the gain $|\lambda_k d_{AHL}|$, where λ_k is defined in Supporting Information Text S1, becomes increasing large. The positions of the system eigenvalues without diffusion are shown with $\times$'s and their limits as the gain approaches infinity are shown with o's. Only the right-most eigenvalues (the ones nearest to the imaginary axis) are shown.

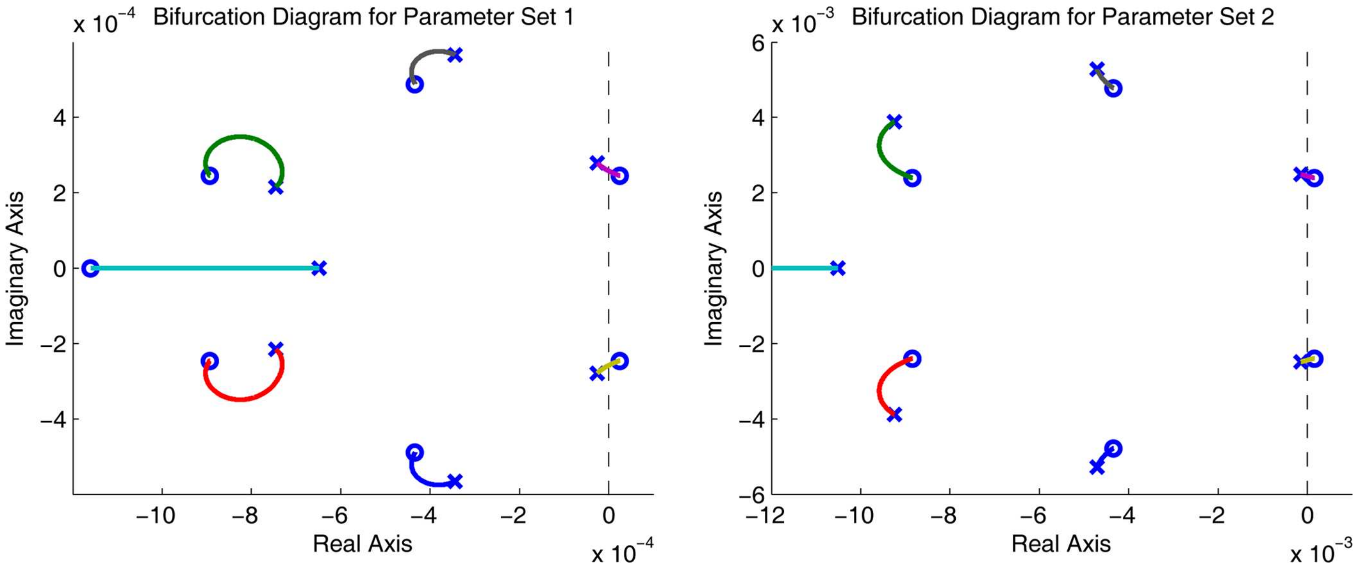

References

[s2] Liu R, Liaw S, Maini P (2007) Oscillatory turing patterns in a simple reaction-diffusion system. Journal of the Korean Physical Society 50: 234–238.
