# Supplementary material for: A Feedback Quenched Oscillator Produces Turing Patterning with One Diffuser
Source: PLoS Comput Biol. 2012 Jan 26;8(1):e1002331. doi: 10.1371/journal.pcbi.1002331 (PMC3266880; doi:10.1371/journal.pcbi.1002331)
Supplement: Text S3 — Choosing parameter values for Parameter Set 1. (PDF) [file pcbi.1002331.s018.pdf]

Text S3 Choosing parameter values for Parameter Set 1

This refers to the parameters given in the "Value for PDE Simulation (Parameter Set 1)" column of Table S1. Accepted literature values were used whenever possible. When literature values were not available, acceptable estimates were made in order to keep the analysis in the realm of experimental plausibility. The parameter values can be split to three groups: known, estimated, and prescribed values. "Known values" are parameters with measured literature values, including protein-DNA dissociation constants, mRNA production rates for given promoters, and promoter leakage levels. These values are considered fixed and cannot be readily changed.

"Estimated values" can be experimentally changed within reasonable ranges, which are also given in Table S1. Half-lives of proteins can be anywhere between minutes to hours and can be controlled by adding or changing their ssrA tags [s12]. Most proteins in our system have half-lives in the tens of minutes. Half-lives of mRNA usually fall in the order of minutes [s14] and can be altered by changing the secondary structure of the mRNA. The half-life of AHL is measured to be approximately 24-48 hours [s5], but can be sped up to the order of minutes in the presence of the enzyme AiiA [s13]. In this study, a steady-state concentration of AiiA is assumed to set the AHL half-life at 15 minutes. The copy numbers for the plasmids was assumed to be low, so a value of 5 is used to represent the averaged plasmid copy number for the complete field of cells.

The "prescribed" parameters consist of the value of constitutively-produced LuxR protein, assumed constant, and the translation rates of mRNA. The steady-state value of a protein can be fixed by adjusting its production rate and degradation rate and should fall in the range between 1 nM to 1 mM in a cell. The translation rate of proteins can be sped up or slowed down by changing the ribosome binding site. This generally yields about 10 proteins per mRNA transcript [s9, 25]. In this analysis, the protein translation rates were the only parameters which were readily changed. Finding protein translation rates for this system to meet the Turing conditions for patterning was the big challenge of this analysis. The known and estimated values created tight constraints for the translation rates.

Due to computational constraints on the stochastic simulations, we restricted our spatial domain to a line of 100 cells ($100\ \mu m$). The literature value for diffusion allowed molecules of AHL to traverse this entire spatial domain very rapidly, obscuring the patterning visually, so we reduced the diffusion constant. Experimentally the diffusion constant will effectively change based on the medium of diffusion, but the same effect can also be achieved by increasing the spatial domain.

References

[s3] Lies M, Maurizi M (2008) Turnover of endogenous SsrA-tagged proteins mediated by ATP-dependent proteases in *Escherichia coli*. Journal of Biological Chemistry 283: 22918–22929.

[s4] Rauhut R, Klug G (1999) mRNA degradation in bacteria. FEMS microbiology reviews 23: 353–370.

[s5] Englmann M, Fekete A, Kuttler C, Frommberger M, Li X, et al. (2007) The hydrolysis of unsubstituted N-acylhomoserine lactones to their homoserine metabolites: Analytical approaches using ultra performance liquid chromatography. Journal of Chromatography A 1160: 184–193.

[s6] Danino T, Mondragón-Palomino O, Tsimring L, Hasty J (2010) A synchronized quorum of genetic clocks. Nature 463: 326–330.

[s7] Elowitz M, Leibler S (2000) A synthetic oscillatory network of transcriptional regulators. Nature 403: 335–338.

[s8] Kierzek A, Zaim J, Zielenkiewicz P (2001) The effect of transcription and translation initiation frequencies on the stochastic fluctuations in prokaryotic gene expression. Journal of Biological Chemistry 276: 8165–8172.
