# Supplementary material for: A Feedback Quenched Oscillator Produces Turing Patterning with One Diffuser
Source: PLoS Comput Biol. 2012 Jan 26;8(1):e1002331. doi: 10.1371/journal.pcbi.1002331 (PMC3266880; doi:10.1371/journal.pcbi.1002331)
Supplement: Text S4 — Reaction set for stochastic simulations. (PDF) [file pcbi.1002331.s019.pdf]

Text S4 Reaction set for stochastic simulations

We developed a set of reactions that, using the law of mass action and the quasi-steady-state approximation, would exactly match our set of PDE equations. The complete reaction set can be found in Table S11 below. For a Hill coefficient of exactly two, we assumed that each promoter had a single binding site to which only the dimerized form of the appropriate activator or inhibitor could bind. The table of reactions follows the species and variable naming scheme used in the PDE set found in (6)-(16) with the addition of dimers p_{x2} and promoters Pr_x. The dissociation constants K_x are combinations of the on and off dimerization and binding constants. In general, $K_x = \sqrt{\frac{k_{\text{doff}_x} k_{\text{off}_x}}{k_{\text{don}_x} k_{\text{on}_x}}}$.

Table S11: Full reaction set for stochastic simulations. Kinetic rate constants chosen to validate quasi-steady-state approximation and to match Parameter Set 2 of Table S1.

**Reaction Type**	**Reactions**
Dimerization	$p_x + p_x \underset{k_{\text{doff}_x}}{\overset{k_{\text{don}_x}}{\rightleftharpoons}} p_{x2}$ for $x \in \{C, L, RA, T\}$
Transcription factor-promoter binding	$Pr_x + p_{x2} \underset{k_{\text{off}_x}}{\overset{k_{\text{on}_x}}{\rightleftharpoons}} Pr_x p_{x2}$ for $x \in \{C, L, RA\}$ $Pr_x + p_{T2} \underset{k_{\text{off}_T}}{\overset{k_{\text{on}_T}}{\rightleftharpoons}} Pr_x p_{T2}$ for $x \in \{TO, TQ\}$
Maximal transcription	$Pr_C \xrightarrow{V_{P_R}} Pr_C + m_L$ $Pr_L \xrightarrow{V_{P_{LlacO-1}}} Pr_L + m_{TO}$ $Pr_{TO} \xrightarrow{V_{P_{LtetO-1}}} Pr_{TO} + m_C$ $Pr_{TQ} \xrightarrow{V_{P_{LtetO-1}}} Pr_{TQ} + m_I$ $Pr_{RA} p_{RA2} \xrightarrow{V_{P_{LuxI}}} Pr_{RA} p_{RA2} + m_{TQ}$
Leaky transcription	$Pr_C p_{C2} \xrightarrow{\ell_{P_R} V_{P_R}} Pr_C p_{C2} + m_L$ $Pr_L p_{L2} \xrightarrow{\ell_{P_{LlacO-1}} V_{P_{LlacO-1}}} Pr_L p_{L2} + m_{TO}$ $Pr_{TO} p_{T2} \xrightarrow{\ell_{P_{LtetO-1}} V_{P_{LtetO-1}}} Pr_{TO} p_{T2} + m_C$ $Pr_{TQ} p_{T2} \xrightarrow{\ell_{P_{LtetO-1}} V_{P_{LtetO-1}}} Pr_{TQ} p_{T2} + m_I$ $Pr_{RA} \xrightarrow{\ell_{P_{LuxI}} V_{P_{LuxI}}} Pr_{RA} + m_{TQ}$
Translation	$m_x \xrightarrow{\epsilon_x} p_x$ for $x \in \{C, I, L\}$ $m_{Tx} \xrightarrow{\epsilon_{Tx}} p_T$ for $x \in \{O, Q\}$
AHL production	$p_I \xrightarrow{v_3} p_I + A$
LuxR-AHL binding	$p_R + A \underset{k_{\text{boff}_{AR}}}{\overset{k_{\text{bon}_{AR}}}{\rightleftharpoons}} p_{RA}$
Degradation	$p_x \xrightarrow{\gamma_x} \varnothing$ for $x \in \{C, T, L, I\}$ $m_x \xrightarrow{\gamma_{mO}} \varnothing$ for $x \in \{C, L, TO\}$ $m_x \xrightarrow{\gamma_{mQ}} \varnothing$ for $x \in \{I, TQ\}$ $A \xrightarrow{\gamma_A} \varnothing$
Diffusion	A in cell $x \xrightarrow{d_{AHL}} A$ in cell $x \pm 1$
