# Supplementary material for: A Feedback Quenched Oscillator Produces Turing Patterning with One Diffuser
Source: PLoS Comput Biol. 2012 Jan 26;8(1):e1002331. doi: 10.1371/journal.pcbi.1002331 (PMC3266880; doi:10.1371/journal.pcbi.1002331)
Supplement: Text S5 — Choosing parameter values for Parameter Set 2. (PDF) [file pcbi.1002331.s020.pdf]

Text S5 Choosing parameter values for Parameter Set 2

This refers to the parameters given in the "Value for Stochastic Simulation (Parameter Set 2)" column of Table S1. With Parameter Set 1 based on physically characterized parts currently used in synthetic biology, we were unable to produce patterning on the stochastic simulator. The goal was then to find a parameter set that deviates slightly from the accepted literature values in order to boost steady-state concentrations in order to produce patterning. All of these values are physically possible based on information in the literature (see references in Table S1) and in the future parts are likely to be found that match our chosen parameter values.

The protein binding affinity, Hill coefficient, and degradation constants in the oscillator loop were made to be equal for all three components as in [s9]. The protein binding affinity is at a concentration of two proteins. Even though the literature values show a binding affinity concentration of less than 1 protein, multiple binding sites and greater than unity plasmid copy numbers mean that the effective binding affinity should be higher than 1 protein. This immediately takes care of the extremely low steady-state value of p_L.

The steady-state concentration of AHL in Parameter Set 1 was too low to develop steep enough gradients necessary for diffusion-driven patterning. It is known that crosstalk exists between different AHL systems. In the deterministic analysis, we used the *V. fischeri* AHL system. The circuitry was changed to a mixed AHL system for stochastic simulation. An AHL synthetase that is unmatched to the *lux* promoter is used. This way the binding affinity of the unmatched AHL to LuxR is much smaller than in the matched case, thereby boosting the steady-state concentration of AHL. The AHL synthetase RhiI from *P. aeruginosa* is one such example of a synthetase that may be used with the *lux* promoter [s10, 11].

Using this realistic but "relaxed" set of parameters, patterns were then observed in the stochastic simulations.

References

[s9] Elowitz M, Leibler S (2000) A synthetic oscillatory network of transcriptional regulators. Nature 403: 335–338.

[s10] Parsek MR, Val DL, Hanzelka BL, Cronan JE, Greenberg EP (1999) Acyl homoserine-lactone quorum-sensing signal generation. Proceedings of the National Academy of Sciences of the United States of America 96: 4360.

[s11] Parsek MR, Schaefer AL, Greenberg EP (1997) Analysis of random and site-directed mutations in rhli, a pseudomonas aeruginosa gene encoding an acylhomoserine lactone synthase. Molecular Microbiology 26: 301–310.
